# HydrogelFinder: A Foundation Model for Efficient Self‐Assembling Peptide Discovery Guided by Non‐Peptidal Small Molecules

**DOI:** 10.1002/advs.202400829

**Published:** 2024-05-05

**Authors:** Xuanbai Ren, Jiaying Wei, Xiaoli Luo, Yuansheng Liu, Kenli Li, Qiang Zhang, Xin Gao, Sizhe Yan, Xia Wu, Xingyue Jiang, Mingquan Liu, Dongsheng Cao, Leyi Wei, Xiangxiang Zeng, Junfeng Shi

**Affiliations:** ^1^ College of Information Science and Engineering Hunan University Changsha 410003 China; ^2^ State Key Laboratory of Chemo/Bio‐Sensing and Chemometrics, School of Biomedical Sciences Hunan University Changsha 410003 China; ^3^ ZJU‐Hangzhou Global Scientific and Technological Innovation Center Hangzhou 311200 China; ^4^ College of Computer Science and Technology Zhejiang University Hangzhou 310013 China; ^5^ Computational Bioscience Research Center (CBRC), Computer, Electrical and Mathematical Sciences and Engineering Division King Abdullah University of Science and Technology (KAUST) Thuwal 23955‐6900 Saudi Arabia; ^6^ Xiangya School of Pharmaceutical Sciences Central South University Changsha 410003 China; ^7^ School of Software Shandong University Jinan 250100 China; ^8^ Joint SDU‐NTU Centre for Artificial Intelligence Research (C‐FAIR) Shandong University Jinan 250100 China

**Keywords:** artificial intelligence, deep generative model, machine learning, self‐assembly

## Abstract

Self‐assembling peptides have numerous applications in medicine, food chemistry, and nanotechnology. However, their discovery has traditionally been serendipitous rather than driven by rational design. Here, HydrogelFinder, a foundation model is developed for the rational design of self‐assembling peptides from scratch. This model explores the self‐assembly properties by molecular structure, leveraging 1,377 self‐assembling non‐peptidal small molecules to navigate chemical space and improve structural diversity. Utilizing HydrogelFinder, 111 peptide candidates are generated and synthesized 17 peptides, subsequently experimentally validating the self‐assembly and biophysical characteristics of nine peptides ranging from 1–10 amino acids—all achieved within a 19‐day workflow. Notably, the two de novo‐designed self‐assembling peptides demonstrated low cytotoxicity and biocompatibility, as confirmed by live/dead assays. This work highlights the capacity of HydrogelFinder to diversify the design of self‐assembling peptides through non‐peptidal small molecules, offering a powerful toolkit and paradigm for future peptide discovery endeavors.

## Graphical abstract

HydrogelFinder, a foundational model for the rational design of self‐assembling peptides, leverages large‐scale non‐peptidal small molecules to navigate chemical space and enhance structural diversity effectively. This approach enables the design of diverse, self‐assembling peptides with low cytotoxicity and biocompatibility, offering a powerful toolkit and paradigm for future peptide discovery endeavors.

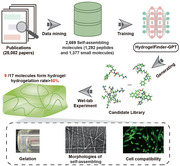

## 1 Introduction

Driven by supramolecular interactions (e.g., hydrogen bonding, hydrophobic interactions, and electrostatic interactions), peptides can self‐assemble in water to form ordered nanostructures, such as nanofibers, which, in turn, form three‐dimensional networks, ultimately leading to supramolecular hydrogelation.^[^ ^1^, ^2^, ^3^, ^4^, ^5^ ^]^ Supramolecular hydrogels resemble extracellular matrices in tissues in that they both have a highly water content and fibrils that function similarly to cytoskeleton. These properties have led to their extensive study as emerging potential biomaterials for tissue engineering,^[^ ^6^ ^]^ drug delivery,^[^ ^7^, ^8^ ^]^ cancer cell inhibition,^[^ ^9^, ^10^ ^]^ regenerative medicine,^[^ ^11^ ^]^ or antibacterial applications.^[^ ^12^ ^]^ Despite these advances in supramolecular hydrogels,^[^ ^13^, ^14^, ^15^, ^16^, ^17^, ^18^ ^]^ designing self‐assembling peptides based solely on molecular structure remains challenging for chemists.^[^ ^19^ ^]^

Numerous classic self‐assembling molecules have been discovered unintentionally rather than through rational design.^[^ ^20^, ^21^, ^22^, ^23^ ^]^ For instance, Weiss et al.^[^ ^23^ ^]^ serendipitously found that cholesteryl 4‐(2‐anthryloxy) butyrate could form a hydrogel while studying its photochemistry. Zhang et al. discovered that a class of amphiphilic peptides derived from the yeast protein, *Zuotin*, could self‐assemble in physiological buffer (e.g., Dulbecco modified Eagle's medium) to form an “insoluble macroscopic membrane”.^[^ ^22^, ^24^ ^]^ Similarly, Xu et al. observed that Fmoc‐D‐Ala‐D‐Ala, an intermediate in peptide synthesis, could form a hydrogel through hydrogen bonding and hydrophobic interactions.^[^ ^25^ ^]^ Many other reports have also described the unexpected discovery of peptides that can self‐assemble into hydrogels under various conditions.^[^ ^23^, ^26^, ^27^, ^28^ ^]^ However, the rational design of self‐assembling peptides using traditional methods faces formidable challenges, particularly in accurately modeling the intricate interactions between water molecules and peptides, and achieving a delicate balance of hydrophilicity and hydrophobicity.^[^ ^29^ ^]^ These further complicate the design of self‐assembly peptides.

In response to these challenges, recent advancements in machine learning have profoundly impacted fields such as chemistry,^[^ ^30^, ^31^ ^]^ materials science,^[^ ^32^, ^33^, ^34^ ^]^ and biomedical research.^[^ ^35^, ^36^, ^37^, ^38^, ^39^, ^40^, ^41^, ^42^ ^]^ Machine learning, as an invaluable tool, has become crucial in deciphering the complexities of peptide self‐assembly. For example, Sankaranarayanan et al.^[^ ^43^ ^]^ have ingeniously harnessed Monte Carlo tree search (MCTS) alongside coarse‐grained molecular dynamics (CGMD) simulations to discovery novel pentapeptides. Wang et al.^[^ ^44^ ^]^ have deftly combined support vector machines (SVM) with CGMD to predict peptides aggregation propensity (AP) and identify potent tetrapeptides. Li et al.^[^ ^45^ ^]^ have employed a robust deep learning framework, along with CGMD, to predict the self‐assembly properties of a vast peptide library, successfully forecasting the AP of both pentapeptides and decapeptides. Despite these advancements, challenges persist in the application of machine learning to peptide self‐assembly discovery. Current methods rely on costly CGMD simulations to derive AP values for peptides in training sets, resulting in a time‐consuming and labor‐intensive process with limitations in generalizing beyond the training data. Moreover, traditional machine learning approaches often focus solely on amino acid sequences, neglecting the significant impact of peptide modifiers on self‐assembly processes within the vast and diverse chemical space of peptides.

**Figure 1:**
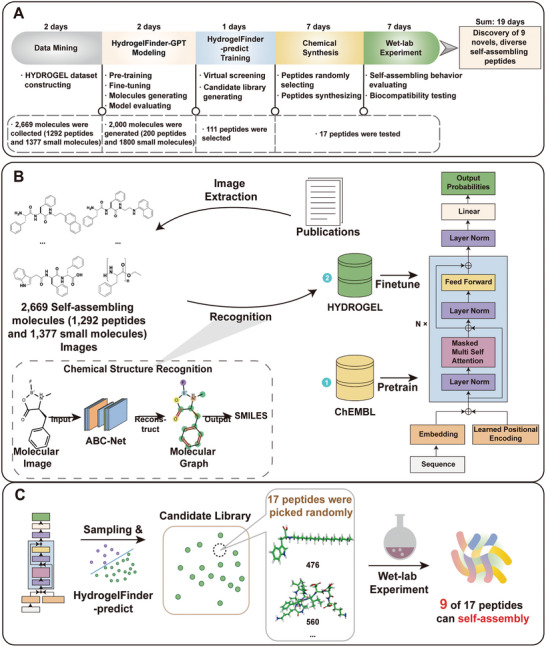
A) Workflow of the artificial intelligence framework for self‐assembling peptide design and discovery. B) The general workflow for the design of self‐assembling peptide using HydrogelFinder‐GPT C) We sampled molecules from the fine‐tuned network, and performed high‐throughput prediction with the HydrogelFinder‐predict to build a candidate library. We randomly selected 17 peptides from the candidate library, 9 of which can self‐assembly under specific conditions.

In this work, we propose HydrogelFinder, an innovative foundation model comprising three key modules: HydrogelFinder‐mining for literature data mining, HydrogelFinder‐GPT employing a deep generative model, HydrogelFinder‐predict as a virtual screening tool (**Figure** **1**). This integrated system facilitates the rational design, rapid production, and screening of self‐assembling peptides. To navigate the complex chemical space of peptides and discover a diverse array of potential self‐assembled peptide candidates, we focus on the perspective of molecular structure, rather than merely through amino acid sequences, to delve into the self‐assembly characteristics of peptides. Leveraging HydrogelFinder‐mining, we construct a molecular library comprising 2669 self‐assembled molecules, encompassing both peptides and non‐peptidal small molecules. Non‐peptidal small molecules play a pivotal role in guiding the exploration of chemical space and enhancing structural diversity. As a proof of concept, utilizing HydrogelFinder‐GPT with this comprehensive library as a training set, we identified 2000 compounds for screening, which yielded 111 previously unreported candidates. Experimental characterization of 17 structurally diverse peptides from this set revealed that nine peptides, ranging from 1–10 amino acids in length, exhibited ability to self‐assemble into hydrogels with diverse properties. Notably, two randomly selected peptides showed low cytotoxicity toward human cell lines. An additional highlight is the identification of the shortest self‐assembling lipid‐peptide compound documented to date that does not require a metal ion. This work establishes HydrogelFinder as a highly effective foundation model for AI‐based design and generation of self‐assembling peptides.

## 2 Results and Discussion

### 2.1 Overview of HydrogelFinder

In pursuit of efficiently sampling structurally diverse self‐assembled peptides within the expansive chemical space, our approach comprises three integral modules. First, HydrogelFinder‐mining engages in literature mining to construct an effective and chemically diverse training dataset. Following this, HydrogelFinder‐GPT employs a deep generative model to model the relationship between molecular structural features and aggregation propensity, and HydrogelFinder‐predict facilitates virtual screening to evaluate candidates.

Specifically, HydrogelFinder‐mining compiles a set of molecular graphs related to self‐assembly, converting these images into SMILES representations.^[^ ^46^ ^]^ This process facilitates the construction of a HYDROGEL‐POSITIVE training dataset, enhancing chemical diversity with a collection of 1292 self‐assembling peptides and 1377 self‐assembling small molecules, totaling 2669 entries (Figure 1A). Additionally, we establish a publicly accessible self‐assembling molecules database for the broader research community at http://hydrogeldb.com. For the rational generation of self‐assembling peptide, we proposed an automated deep generative model, HydrogelFinder‐GPT (Figure 1B). This model, utilizing a transformer architecture, learns the rules of self‐assembly from molecule sequence strings.^[^ ^47^ ^]^ Beginning with pretraining on a comprehensive collection of Chembl small molecules to grasp molecular grammar, the model undergoes fine‐tuning on an autonomously constructed HYDROGEL‐POSITIVE dataset (Table S2, Supporting Information). This fine‐tuning process refines the model's understanding towards self‐assembly properties. Candidates generated by HydrogelFinder‐GPT undergo evaluation through HydrogelFinder‐predict before experimentational validation.

### 2.2 Generation of Self‐Assembling Molecules by HydrogelFinder‐GPT

**Figure 2:**
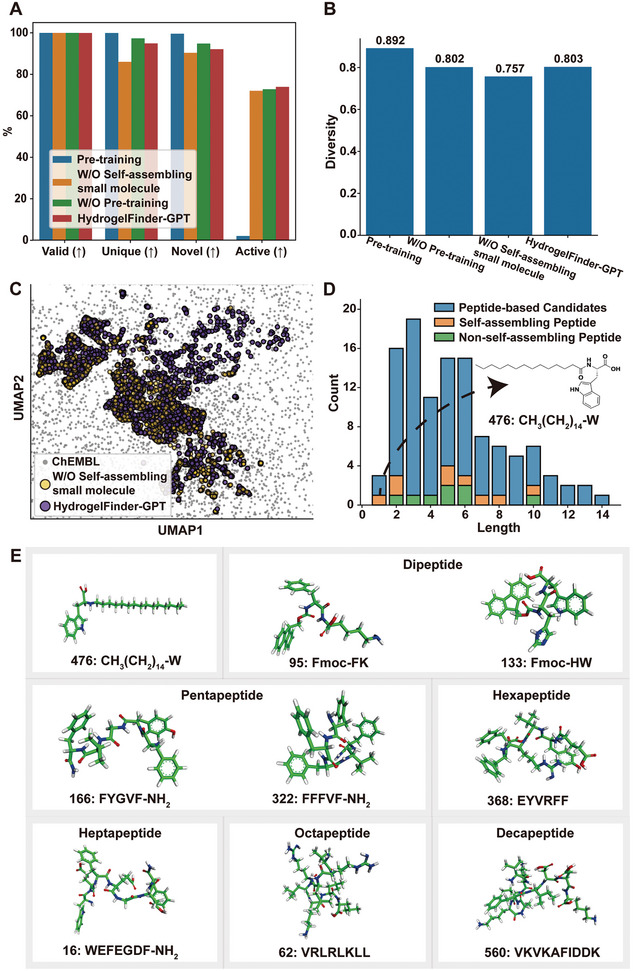
Performance evaluation of HydrogelFinder‐GPT and chemical structures of self‐assembling peptides. A) Performance comparison of generative models with different training strategies over valid, unique, novel and active on generation task (Details see Section 4.2 and Table 1). B) The generative capacity of model structural diversity under different training strategies. C) UMAP visualization of the chemical space distribution of candidates generated by HydrogelFinder‐GPT and generated by training set without self‐assembling small molecules. D) The statistical distributions of peptide‐based candidates. The peptide‐based candidates have 111 sequences (blue), of which 9 molecules can self‐assembly (Orange), while 8 molecules failed (Green). E) Chemical structures and identification numbers (IDNs) of the nine peptides selected from the candidate library that are able to self‐assembly.

To evaluate the efficacy of self‐assembling molecule generation by HydrogelFinder‐GPT, we conducted a comprehensive evaluation of the model's performance based on validity, uniqueness, novelty, and activity under various training strategies. The model‐generated sequences were subjected to evaluation using HydrogelFinder‐predict, with the active rate, representing the ratio of potential self‐assembly, serving as a key metric. As shown in **Figure** **2**A, HydrogelFinder‐GPT achieved the highest active rate at 73.98%, surpassing other training strategies. Additionally, HydrogelFinder‐GPT exhibited impressive figures of 94.95% uniqueness and 92.15% novelty, signifying its capability to produce de novo and valid molecules even after the finetuning process (see details in Section 4.2 and **Table** **1**).

**Table 1:** Performance of generative neural network (Figure 2A Data Supplement).

Metrics	Pre‐training	W/O Self‐assembling Small Molecule	W/O Pre‐training	HydrogelFinder‐GPT
Valid (↑)	100%	100%	100%	99.95%
Unique (↑)	99.95%	86.05%	97.40%	94.95%
Novel (↑)	99.62%	90.41%	94.87%	92.15%
Active (↑)	1.99%	72.05%	72.83%	73.98%

Having established the model's competence in generating self‐assembling molecules, we next investigated properties associated with gelation ability, such as hydrogen bond acceptors (HBA) and donors (HBD), number of basic groups (nBase), Ghose–Crippen LogKow (LogP), topological polar surface area (TPSA), and molecular weight (Mol.wt),^[^ ^48^ ^]^ these values were determined through RDKit calculations. We randomly sampled chemicals of the same size (2000 sequences) from both the HYDROGEL‐POSITIVE and HYDROGEL‐NEGATIVE datasets, plotting the distributions for each parameter (Figure S1, Supporting Information). The analysis revealed that the distribution of de novo candidates for each property closely mirrored that of the HYDROGEL‐POSITIVE dataset, further substantiating the effectiveness of HydrogelFinder‐GPT in designing self‐assembly‐like compounds (More details in the Supporting Information).

### 2.3 Exploration of Structurally Diverse Self‐Assembling Peptides by HydrogelFinder‐GPT

To achieve structurally diverse self‐assembling peptides, our strategy involved the guidance of chemical space exploration using non‐peptidal small molecules. To assess the diversity of candidates generated by the model under different training strategies, we employed the Tanimoto similarities as a metric (More details in Methods). As shown in Figure 2B, HydrogelFinder‐GPT generated candidates with a high diversity score of 0.803. This notable diversity is attributed to the incorporation of non‐peptidal small molecules during training, its removal resulted in a decreased diversity of the generated candidates to 0.757. Additionally, we visualized the model output features using uniform manifold approximation and projection (UMAP) plots.^[^ ^49^, ^50^ ^]^ As shown in Figure 2C, the candidates generated by HydrogelFinder‐GPT exhibited a wider chemical space distribution compared to the training set without small molecules. This result underscores the significant enhancement in our model's performance with the addition of non‐peptidal small molecules data to the training set.

As a proof of concept, we present the length and decoration statistic for 111 peptide‐based candidates. In Figure 2D, our model demonstrated the ability to generate sequences with lengths ranging from 1 to 14 amino acids, surpassing the length of pentapeptides reported in previous studies^[^ ^19^, ^43^, ^44^, ^45^, ^51^ ^]^ The Orange bars represent the self‐assembling peptides confirmed in subsequent wet‐lab experiments, while the green bars represent those that did not exhibit self‐assembly (details in Section 2.6). Specifically, we highlight the successful self‐assembly of nine peptides, comprising amino acid sequences of seven distinct lengths (Figure 2E). Notably, we reported the discovery of the shortest self‐assembling lipid peptide, gel **476**, which comprises only a single amino acid with a long alkyl chain.

### 2.4 Discovery of Self‐Assembling Peptide Derivatives by HydrogelFinder

An often‐overlooked challenge in the development of self‐assembling peptides lies in understanding the influence of chemical modification. To address this, we selected gel **476** and gel **133** as case studies, representing successful instances of self‐assembling peptides with chemical modification identified through our studies. In our study, we compared activity scores of gel **476** and gel **133** with and without modification (obtained by HydrogelFinder‐predict and RDKit). Additionally, we scrutinized a range of properties associated with gelation ability, as summarized in **Table** **2**. The results showed that a significant decrease in the active rate, LogP, and molecular weight of the peptides after the removal of modification. This decline can be attributed to the fact that these modifications can alter the hydrophilic and hydrophobic nature of the peptides. For example, the addition of 9‐fluorenylmethyl carbamate (Fmoc) group increases hydrophobicity, potentially facilitating the self‐assembly of peptides under certain conditions. Moreover, the modification group may introduce new intermolecular interactions such as hydrogen bonding, hydrophobic interactions, which are critical for the self‐assembly.

**Table 2:** Computational characterization and activity of peptides with and without modifiers.

Sequence	Active	LogP	HBA	HBD	NBASE	TPSA	M.W
CH_3_(CH_2_)_14_‐W	1	5.9809	2	3	0	82.19	414.288
W	0.703	1.1223	2	3	1	79.11	204.089
Fmoc‐HW	1	4.1529	5	5	0	149.2	563.216
HW	0.003	0.5729	4	5	1	136.8	341.144

**Figure 3:**
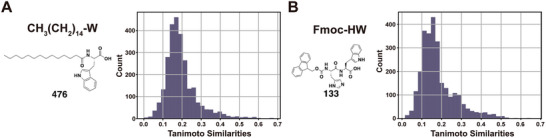
Structures of self‐assembling peptides A) 476 and B) 133 with modification and corresponding distributions of Tanimoto similarities to HYDROGEL‐POSITIVE data set.

To further demonstrate the model's proficiency in discovering structurally diverse self‐assembling peptides, we computed the Tanimoto similarities of gel **476** and gel **133** to the HYDROGEL‐POSITIVE dataset (**Figure** **3**). The majority of compounds in the training set exhibited substantially dissimilar from gel **476** and gel **133**, with mean Tanimoto similarities of 0.19 and 0.18, respectively. This serves as additional evidence of HydrogelFinder's ability to identify structurally diverse self‐assembling peptides with modification.

### 2.5 Evaluation of Gelation Behavior

As shown in Figure 1C, we next sought to experimentally validate the performance of our AI method. To this end, we randomly selected 17 peptides for synthesis, spanning a range of HydrogelFinder‐predicted scores from low to high. Notably, these peptides are entirely new and do not appear in the HYDROGEL‐POSITIVE dataset. We subsequently evaluated their gelation capability (Figure S2, Supporting Information). After purification by HPLC, the obtained compounds were dissolved in water and the pH of the solution was adjusted to trigger self‐assembly and hydrogelation. Non‐self‐assembling peptides formed either a precipitate or a low viscosity fluid irrespective of pH (2–12). Nonetheless, more than half of these peptides (9/17) could self‐assemble in aqueous solution. This high proportion of self‐assembling peptides confirmed the predictive accuracy of the HydrogelFinder model. The gelation properties of the nine self‐assembling peptides are summarized in **Table** **3**.

**Table 3:** Hydrogelation properties of selected molecules.

IDN	Sequence	M.W.	Calc. pI^a)^	Conc.	pH	Buffer	Images
**16**	WEFEGDF‐NH_2_	927.4	2.9	1.0 wt%	5.0	1X PBS	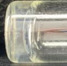
**62**	VRLRLKLL	1009.7	12.4	1.0 wt%	8.0	1X PBS	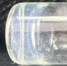
**95**	Fmoc‐FK	515.2	10.1	2.0 wt%	3.0	Water	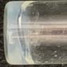
**133**	Fmoc‐HW	563.2	7.9	1.0 wt%	3.0	Water	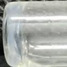
**166**	FYGVF‐NH_2_	630.7	7.	0.5wt%	7.0	Water	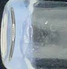
**322**	FFFVF‐NH_2_	704.3	7.0	1.0 wt%	7.0	Water	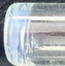
**368**	EYVRFF	859.4	6.9	1.0 wt%	3.0	1X PBS	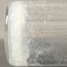
**476**	CH_3_(CH_2_)_14_‐W	414.3	2.5	3.0 wt%	5.0	Wtaer	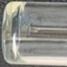
**560**	VKVKAFIDDK	1161.4	9.8	1.0 wt%	8.0	1X PBS	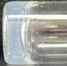

^a)^ Calc. pI is obtained from: https://www.novopro.cn/tools/calc_peptide_property.html

Observing gel behavior in an inverted test tube is a rapid and facile approach to determine whether a dissolved peptide formed a gel. Representative images of the nine candidates holding water and resisting flow, which together indicated gel formation, are shown Table 3. Interestingly, we observed that all peptides containing proline (P) failed to form a hydrogel (Table S2, Supporting Information). Additionally, only a few self‐assembling peptides composed of proline were detected in the hydrogel‐positive database. This suggested that the presence of proline reduces conformational flexibility, diminishing the likelihood of hydrogel formation. Obviously, the pH value of each hydrogel ranged widely, between 3.0 (highly acidic) to 8.0 (weakly basic), and these pH values did not exactly match their calculated isoelectric points (pI). It also warrants mentioned that molecules **95** and **133** shared the same Fmoc motif, which is commonly used to protect amino acids during solid‐phase peptide synthesis. Additionally, peptide **368** formed an opaque hydrogel, likely attributable to large aggregates in the hydrogel matrix. Furthermore, previous reports demonstrated that aromatic‐aromatic interactions between Fmoc moieties can promote the hydrogelation of small molecules.^[^ ^52^ ^]^ Interestingly, as a lipid peptide, molecule **476**, comprised of a single amino acid could also form a gel at pH 5.0, thus representing the shortest lipid peptide of which we are aware.

### 2.6 Biophysical Characterization of Self‐Assembling Peptides

**Figure 4:**
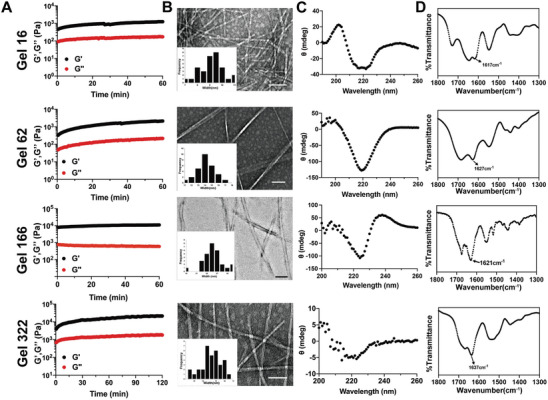
Biophysical characterization of hydrogel 16, 62, 166, 322. A) Rheological dynamic time sweep monitors the storage modulus (G’) and loss modulus (G’’) of different hydrogels as a function of time B) Representative TEM images reveal the morphologies of selected hydrogels, insets are distribution of nanofibers width (scale bar = 100 nm). C) CD spectra of hydrogels suggested the formation of *β*‐sheet structure. D)FT‐IR spectra of hydrogels further confirmed their specific secondary structures.

To further explore the gelation properties and self‐assembly behavior of the candidate self‐assembling molecules, we carried out a series of biophysical characterization experiments. The rheological properties of a hydrogel can vary in a manner dependent on their structure, and moreover, these properties are typically critical for their function in tissue engineering, drug delivery, or other applications. An oscillatory rheological analysis was performed to monitor hydrogel storage modulus (G′, a measure of the elastic response of the material) as a function of time (**Figure** **4**A), revealing a gradual increase in gel **16**, reaching equilibrium at 1254 Pa, and with a consistently higher storage modulus than loss modulus (G″, a measure of viscosity), suggesting the formation of a robust hydrogel. Self‐assembling peptide **62**, **166**, and **322** exhibited a similar trend, reaching equilibrium at 2,143^ ^Pa, 1,0991^ ^Pa, and 21,393^ ^Pa, respectively, which were all consistently higher than their loss moduli, implying that these peptides could form relative rigid hydrogels.

TEM was then used to examine morphology of hydrogel matrix, which revealed the formation of 9–10 nm width nanofibers in gel **16**, while the self‐assembled nanofibers in gels **62**, **166**, and **322** were approximately 14 nm, 25 nm, and 14 nm, respectively. These nanofibers were entangled with one another, forming a 3D‐network that comprised the hydrogel matrix (Figure 4B).

Circular Dichroism (CD) spectroscopy is a valuable technique for detecting secondary structures in the peptide assemblies. In the Far‐UV range (190–260 nm), the majority of chromophores were peptide bonds.^[^ ^53^ ^]^ The CD spectrum of hydrogel **16** had a broad negative band at 218 nm, which was typical of a *β*‐sheet conformation. Similar features were detected in gels **62**, **166** and **322**, suggesting the prevalence of *β*‐sheets in the self‐assembling peptides, which agreed well with fact that the majority of self‐assembling molecules adopted *β*‐sheet conformation.^[^ ^54^ ^]^ To further explore the molecular configuration of the assembled structures, we used Fourier‐transformed infrared spectroscopy (FT‐IR) to examine each gel.^[^ ^55^, ^56^ ^]^ In gel **16**, a peak was detected at 1617 cm^−1^ which was assigned as an amide I band, suggesting the formation of a *β*‐sheet. Similarly, a peak at 1627 cm^−1^ was observed in gel **62**, a strong peak at 1621 cm^−1^ was present in gel **166** spectra, and gel **322** also had a peak at 1637 cm^−1^, all of which indicated that a β‐sheet conformation was adopted by the peptides during gelation. Overall, these data consistently supported the likelihood that these peptides self‐assembled into ordered nanostructures which formed *β*‐sheets that comprised nanofibers in a gel matrix.

### 2.7 Biocompatibility of Self‐Assembling Peptides

**Figure 5:**
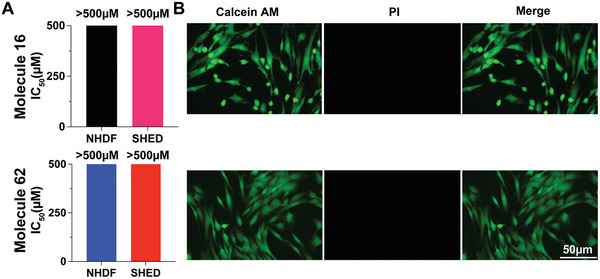
Cell compatibility analysis of compound 16 and 62. A) IC50 of NHDF and SHED cells treated with molecule 16 (top panel) and 62 (bottom panel) for 24 h. B) Live/dead assays of cells grown on the surface of hydrogel 16 (right of top panel) or 62 (right of bottom panel) for 24 h showing high biocompatibility with SHED cultures. Living cells show positive staining with calcein AM (green), while dead or compromised cells are stained with propidium iodide (PI, red). Each column represents three individual experiments (n = 3).

Supramolecular hydrogels can serve as scaffolds for cell culture because of their high similarity with an extracellular matrix.^[^ ^57^ ^]^ However, this application requires high biocompatibility. Thus, we chose to evaluate cytotoxicity of peptides **16** and **62** using MTT assays. As common cell lines, human dermal fibroblasts (NHDF) and stem cells from human exfoliated deciduous teeth (SHED) were chosen as model cells for evaluating the cytotoxicity of peptides,^[^ ^69^, ^70^ ^]^ peptides **16** and **62** exhibited high cell compatibility at concentrations as high as 500 µM (**Figure** **5**A). Live/dead assays using calcein AM to stain for viable cells and propidium iodide (PI) to stain for compromised or dead cells cultured on the gel surface showed that the vast majority of SHED cells were positive for calcein AM staining, while few or no PI stained cells could be detected (Figure 5B). These results implied that either molecule itself or the bulk gel was exhibited limited toxicity, suggesting its potential use in cell culture applications.

## 3 Discussion

We developed HydrogelFinder, a foundation model integrating literature mining module, a generative language model, and machine learning for de novo design of self‐assembling peptides. This model was complemented by comprehensive experimentally validation, confirming gelation behavior and biocompatibility. In essence, HydrogelFinder was devised to construct a candidate library and establish a publicly accessible database for the hydrogel research community (http://hydrogeldb.com). It leverages non‐peptidal small molecules to guide the *in‐silico* discovery of structurally diverse self‐assembling peptides. Through experimental characterization of 17 randomly selected, structurally diverse candidate peptides, we identified nine molecules capable of forming hydrogels in water under different conditions. These peptides displayed distinct self‐assembly behaviors and length distributions spanning from 1 to 10 amino acids, with two modification influencing peptide self‐assembly. Biophysical characterization revealed the formation of ordered nanostructures, such as nanofibers, within the hydrogel matrix. Notably, two hydrogels demonstrated low cytotoxicity in vitro, suggesting potential applications as scaffolds for cell culture or drug delivery.

Furthermore, the extensive hydrogel candidate library generated in this work remains largely unexplored, holding the potential to address ongoing and future research questions. This includes robust determination of the common structural features defining self‐assembling molecules. Recognizing that the Transformer‐based architecture excels in extracting 1D sequence information, yet falls short in capturing the 3D complexities of peptide chain's secondary, tertiary and quaternary structures. To address this issue, we are actively developing geometric deep learning models. These models aim to efficiently extract 3D features and fuse multimodal information, enhancing the accuracy and efficiency of candidate molecule generation.

This work thus establishes a powerful framework for designing self‐assembling peptides, accompanied by a sizable database for public exploration and the experimental characterization of several hydrogels. Beyond a proof‐of‐concept demonstration, HydrogelFinder stands poised for use in the design and screening of new peptide crucial for urgent biomedical applications.

## 4 Experimental Section

### Datasets

The original data used in this work were originated from a few pre‐available sources (ChEMBL, CPPsite, and ZINC). Data from ChEMBL was used to pre‐training generative neural network (HydrogelFinder‐GPT pre‐training network). Literature data mining by HydrogelFinder‐mining to construct HYDROGEL‐POSITIVE dataset for HydrogelFinder‐GPT fine‐tuning. CPPsites and ZINC was used to construct a HYDROGEL‐NEGATIVE dataset. Training the HydrogelFinder‐predict model using HYDROGEL‐POSITIVE and HYDROGEL‐NEGATIVE dataset.

ChEMBL. ChEMBL^[^ ^58^ ^]^ was a large molecules database. The pre‐training network was trained with a subset of ChEMBL version 25. Initially, the complete dataset was standardized with the MolVS Python module using the super parent setting, which standardizes fragment, charge, isotope, stereochemistry and tautomeric states. Molecules were filtered to only contain the atoms [H, C, N, O, F, S, Cl, Br] and heavy atoms that were fewer than 50 in number. In general, a subset of ChEMBEL was constructed for pretraining procedure with 300000 molecules.

CPPsite. CPPsite^[^ ^59^, ^60^, ^61^ ^]^ was an updated version of manually curated database (CPPsite) of cell‐penetrating peptides (CPPs). The current version holds around 1850 peptide entries, including their predicted tertiary structure of cell‐penetrating peptides. CPPsite also maintains information on cell‐penetrating peptide properties and abilities to delivery different cargo in model systems (in vitro and in vivo). In this work, CPPsite was only used as a part of negative samples in the HYDROGEL dataset.

ZINC. ZINC^[^ ^62^ ^]^ was a free database of commercially‐available compounds for virtual screening. ZINC contains over 230 million purchasable compounds in ready‐to‐dock, 3D formats. ZINC also contains over 750 million purchasable compounds one can search for analogs in under a minute. In this work, “aggregator” was used as the filter key to search this database, and used the result as another part of negative samples in the HYDROGEL dataset. It was worth to note that “aggregates” typically denotes the non‐specific, often irreversible association of molecules, which frequently leads to the formation of amorphous structures. In contrast, “self‐assembly” refers specifically to the process by which molecules organize themselves into ordered, functional structures through non‐covalent molecular interaction.^[^ ^63^, ^64^ ^]^ While there can be overlap between aggregation and self‐assembly in some cases, known aggregators were chosen to use as a negative training set for HydrogelFinder‐predict to ensure that the model prioritizes the prediction of self‐assembly behavior.

HYDROGEL dataset. For the HYDROGEL‐POSITIVE dataset construction, a thorough search of the Pubmed database was conducted using the “hydrogel” as a query, which yielded 25082 publications. Embedded images in relevant papers were extracted for the training set using the pdf2image python library. Each image was then enumerated, and the molecular graph within each image was identified and translated into SMILES strings using ABC‐Net,^[^ ^65^ ^]^ an advanced model for chemical structure recognition tasks developed by our group. According to the findings in the papers, compounds used to successfully generate hydrogels were classified as HYDROGEL‐POSITIVE samples, while other compounds were classified as HYDROGEL‐NEGATIVE samples. All molecules in the HYDROGEL‐POSITIVE dataset were thus considered self‐assembling molecules, which enable hydrogel formation. Apart from the data collected in publications, the HYDROGEL‐NEGATIVE dataset also included compounds obtained from publicly accessible databases, such as ZINC and CPPsites. Data filtering was conducted using the RDKit python library to exclude compounds that failed to transform into a corresponding molecular graph in the HYDROGEL dataset (Supporting Information). In total, the HYDROGEL dataset contained 2669 positive samples and 16761 negative samples after removing duplication, 70% of which were then randomly selected for the training set, while the remaining 10% was used as the testing set. During the training process, 20% of the training set was set aside to serve as the validation set (Table 3).

### HydrogelFinder‐GPT

A Transformer decoder model was used as architecture for our training, taking the sequence as input using one‐hot encoding. The method consists of two parts: pre‐training and fine‐tuning.

*Model pre‐training*: The decoder module was used as our basic attention module (Figure 1B). In general, this network contains 18 decoder modules, each of which has 48 dimensional states, 12 attention heads and a position‐wise feed‐forward network with 512 dimensional inner states. Here, the chemical generation task was modeled as a text generation task in the general natural language processing domain. Specifically, given an unsupervised dataset of compounds *v* = {c_0_,…, c_m_}, a pair of tokens were inserted ([START] and [END] token) for each compound, and concatenated them to get our pretraining corpus $U=\{u_{0},\text{…},u_{n}\}$, where m denotes the size of the dataset and *n* was the number of tokens in the corpus. A standard language modeling objective was used to maximize the following likelihood:

(1) $$L\left(u\right)=\sum_{i}\log\;P\left(u_{i}\left|u_{i-k},\text{…},\right.u_{i-1};\Theta \right)$$

where *k* was the size of the context window, and the conditional probability *P* was modeled using a neural network with parameters Θ. In this work, *k* was set to 128. The model trained on 40GB NVIDIA V100 in 36 hours.

*Model Performance Evaluation*: The testing set with 271 samples of HYDROGEL‐POSITIVE dataset was used for the final performance assessment of HydrogelFinder‐GPT. The metrics, included validity,^[^ ^66^ ^]^ uniqueness,^[^ ^66^ ^]^ novelty,^[^ ^66^ ^]^ FCD,^[^ ^67^ ^]^ nearest neighbor similarity (SNN),^[^ ^66^ ^]^ Fragment similarity (Frag),^[^ ^68^ ^]^ scaffold similarity (Scaff),^[^ ^69^ ^]^ Active and diversity. Among them, the valid percentage of molecular strings that can be translated back into molecular graphs; the unique percentage of non‐duplicated molecular strings; and the novel percentage of chemicals that were not present in the training set. FCD measures the similarity of chemical structures and bioactivities between a testing set and the generated chemicals according to features extracted by a well‐trained deep neural network.^[^ ^67^ ^]^ Frag and Scaff were cosine distances between the vectors of fragments or scaffold frequencies correspondingly to a generated distribution and the distribution of a testing set. SNN was the average similarity of generated chemicals to the nearest chemical from a testing set distribution. The four metrics were implemented by the MOSES.^[^ ^67^ ^]^ A sample scored higher than 0.5 in the HydrogelFinder‐predict was considered as a candidate capable to form a hydrogel. The active metric represents the average score between the active proportion of candidates in novel chemicals and in total generated chemicals. The diversity of a set of molecules were define as the average pairwise Tanimoto similarities between them, where Tanimoto similarities *dist* (*X*, *Y*) =  1 − *sim*(*X*, *Y*).

The performance was assessed of the model using several metrics, including SNN, Frag, FCD, and Scaff, to gauge the similarity between the generated chemicals and the hydrogel data in the testing set of the HYDROGEL‐POSITIVE dataset from different angles (**Table** **4**). Notably, HydrogelFinder‐GPT significantly outperforms the pre‐training network across these metrics. Only in cases where small molecules were absent from the training set did the SNN and Scaff metrics favor the pre‐training network. However, overall, HydrogelFinder‐GPT exhibited a more balanced performance. The data generated by HydrogelFinder‐GPT closely resembled the testing set, implying that the model effectively learned to characterize the distribution of hydrogels within the chemical space and can generate chemically similar yet completely new compounds.

**Table 4:** Performance of generative neural network (the up‐arrows denote that the higher scores are considered better, the down‐arrows mean that lower scores are better).

Metrics	Pre‐training	W/O Self‐assembling Small Molecule	W/O Pre‐training	HydrogelFinder‐GPT
FCD (↓)	33.7017	7.1341	5.3607	4.9586
SNN (↑)	0.2153	0.5936	0.4730	0.5149
Frag (↑)	0.6705	0.9952	0.9947	0.9957
Scaff (↑)	0.1388	0.5576	0.4866	0.4821

*High‐throughput Prediction HydrogelFinder‐predict Model*: To evaluate the performance of the generative neural network to generate potential self‐assembling compounds, a probabilistic SVM classification model was used (More details in Supporting Information). The model was trained to discriminate active compounds that could self‐assemble to form hydrogels from inactive ones according to their 2048‐bit‐radius extended connectivity fingerprint (ECFP) representations. Given the size of the HYDROGEL‐POSITIVE datasets and the HYDROGEL‐NEGATIVE datasets were highly imbalanced (Table S2, Supporting Information), Sampling was performed up to resample positive samples with respect to the negative ones until they reach the same size (See method in the Supporting Information). The model with C = 10 and γ = 0.01 was considered to have the highest AUROC (0.9862) toward the testing set of the HYDROGEL dataset.

### Peptides Synthesis

All peptides were synthesized via standard solid‐phase peptide synthesis using a CS136S peptide synthesizer, with Rink‐AM resin or 2‐Cl resin and activation by HCTU. The resin‐bound peptides were cleaved using a cocktail of TFA/Triisopropylsilane/H_2_O (95:2.5:2.5) for 3 h. The resin mixture was filtered and washed with excess TFA. Crude peptides were obtained by concentrating the filtrate and precipitating it with cold ether. The crude product was purified by reverse phase HPLC with a semi‐preparative C18 column. HPLC solvents comprise solvent A (0.1% TFA in MilliQ water) and solvent B (0.1% TFA in 9:1 acetonitrile/water). All peptides were lyophilized after HPLC purification, and subsequently analyzed using analytical HPLC and MALDI‐TOF MS.^[^ ^18^ ^]^

### Preparation of Hydrogel

All compounds were placed in a glass tube (diameter 10 mm) and first dissolved in D.I water, sonicated for 5 min and putted on ice for 30 min. Then added 2x PBS buffer (5.4 mM KCl, 20 mM Na_2_HPO_4_, 4 mM KH_2_PO_4_) or D.I water to reach the final concentration of 1.0 wt%. pH was adjusted with NaOH or HCl. The solutions were stored in 37˚C incubator overnight. Gelation was confirmed by the inverting method. In this method, when peptide solution had already formed a gel at the bottom of sample vial, the vial was inverted, and the gel remained in place without falling or flowing.

### Circular Dichroism Spectroscopy

Circular Dichroism spectra were collected on Jasco X spectropolarimeter (Jasco corp., Tokyo, Japan). CD wavelength spectra were measured from 260 to 200 nm using a 0.1 mm quartz cell. Wavelength scans were collected by scanning in 1 nm step intervals with a 3s averaging time.

### Oscillatory Rheology

All rheological experiments were performed on an Anton Parr equipped with a steel 15 mm parallel geometry tool. In a typical time‐dependent experiment, peptide solution was transferred to the rheometer stage and lower the geometry to 0.5 mm, then the temperature was increased to 37˚C within 1.0 min. To avoid dehydration, a layer of silicon oil was applied around the edge of the sample at the start of the measurement. Dynamic strain sweep experiments were performed to ensure that the time‐sweep data was collected in the linear regime of strain. The dynamic strain sweep was performed varying the strain from 0.1 to 100% at a constant frequency (6 rad s^−1^).

### Transmission Electron Microscopy

The sample was prepared by placing a drop of peptide solution on a 200‐mesh copper grid (Electron Microscopy China) and allowed to stand for 1.0 min, then blotted with filter paper. Subsequently a drop of 1.0% Uranyl Formate was placed on the grid and allowed to stand for 1–2 min, then blotted with a piece of filter paper and left to air dry. Images were taken with a JEOL JEM‐2100Plus at 80 kV accelerating voltage. By calculating the width of peptide, The image J was used to measure 30 times for 1 TEM picture, and to gather statistics with frequency.^[^ ^26^ ^]^

### Fourier Transform Infrared Spectroscopy (FTIR)

Hydrogel sample was prepared for FTIR studies at a concentration of 1.0 wt% in PBS buffer or D.I water. Prepared hydrogel was lyophilized and dried hydrogel (xerogel) powder was embedded in KBr pellet and analyzed in FTIR. The spectrum was collected using a Nicolet In MX microscopic infrared spectrometer (Thermo Scientific Co., USA) between the wavelengths 4000–400 cm^−1^ under 16 scans on an average. KBr thin film was used as blank control.^[^ ^70^ ^]^

### Cell Viability Assay

MTT assay was employed to assess cytotoxicity of all molecules. In a typical experiment, NHDF cells were seeded into 96‐well plate at a density of 8000 cells/well, allowed to adhere overnight at 37˚C, 5% CO_2_. The culture medium was replaced with fresh serum‐free medium containing 0.1–500 µM peptides. Blank medium or DMSO was used as positive control and negative control, respectively. After 48 h incubation period, 100 µL of fresh serum‐containing media was added into each well. 10 µL of (3‐(4,5‐Dimethylthiazol‐2‐yl)−2,5‐diphenyl‐tetrazolium bromide (MTT, 5 mg mL^−1^ in PBS) was added to each well and samples incubated for 3–4 hours, then the medium was replaced with 100 µL DMSO and incubated at 37˚C with shaking for 0.5‐1 h to facilitate formazan crystal solubilization. Absorbance was recorded at 540 nm using a UV microplate reader (Molecular Devices, Spectra Max M5). The absorbance of the negative controls was subtracted from each sample as a blank, and the percent viability was calculated as follows: (Absorbance peptide‐treated cells – Absorbance negative controls) / (Absorbance untreated cells – Absorbance negative controls) × 100. IC_50_ was calculated using Graphpad Prism 9.0 software.^[^ ^71^ ^]^

### Biocompatibility Test

Following the protocol in previous work,^[^ ^13^ ^]^ hydrogel (1.0 wt%, 50 µL) was prepared in a 96 well plate. The plate was placed into an incubator at 37˚C and 5% CO_2_ and allowed to equilibrate for 24 h. Serum‐free MEM‐α media (Gibco)of 100 µL was added on the top of gel and equilibrate overnight. Stem cell from human exfoliated deciduous teeth was trypsinized and counted using a hemocytometer. The resulting suspension was diluted with serum containing DMEM, 100 µL cell suspension (8,0000 cells mL^−1^) was placed onto the top of hydrogel. After 24 h incubation, the medium was removed and washed gently with PBS to remove the serum proteins. Cell viability was evaluated by using a Live/dead assay. Typically, 100 µL assay buffer containing both 1 µL calcein AM and 1 µL PI was added into each well. The dye was allowed to incubator for 30 min before washing with PBS 3 times, after that 100 µL cell imaging solution was added into each well for imaging. Fluorescence images were taken on EVOS FL Auto.^[^ ^60^ ^]^

### Statistics

All quantitative statistical experiments were replicated at least three times(n = 3). Data were presented as mean ± standard deviation (X ± SD). Statistical analyses were performed in GraphPad Prism 9 software.

## Conflict of Interest

The authors declare no conflict of interest.

## Author Contributions

X.R., J.W., and X.L. contributed equally to this work. J.S. and X.Z. conceived the original ideas and guided the project. X.L., X.R. and J.W. designed and performed the experiments, and analyzed the data. X.G., K.L., Y.L., Q.Z., L.W and D.C. guided the design of computing algorithm. S.Y. helped in sample collection. X.W. and X.J did part of wet laboratory experiments. M.L constructed the website. All authors provided critical feedback and helped to shape the research, analysis and manuscript.

## Supporting information

Supporting Information

## Data Availability

The data that support the findings of this study are available from the corresponding author upon reasonable request.
