# Supplementary material for: HydrogelFinder: A Foundation Model for Efficient Self‐Assembling Peptide Discovery Guided by Non‐Peptidal Small Molecules
Source: Adv Sci (Weinh). 2024 May 5;11(26):2400829. doi: 10.1002/advs.202400829 (PMC11234452; doi:10.1002/advs.202400829)
Supplement: Supplementary file 1 — Supporting Information [file ADVS-11-2400829-s001.pdf]

ADVANCED SCIENCE
Open Access

Supporting Information

for *Adv. Sci.*, DOI 10.1002/advs.202400829

HydrogelFinder: A Foundation Model for Efficient Self-Assembling Peptide Discovery Guided by Non-Peptidal Small Molecules

Xuanbai Ren, Jiaying Wei, Xiaoli Luo, Yuansheng Liu, Kenli Li, Qiang Zhang, Xin Gao, Sizhe Yan, Xia Wu, Xingyue Jiang, Mingquan Liu, Dongsheng Cao, Leyi Wei, Xiangxiang Zeng and Junfeng Shi**

Supplementary Material

HydrogelFinder: A Foundation Model for Efficient Self-assembling Peptide Discovery Guided by Non-peptidal Small Molecules

Xuanbai Ren[1,#], Jiaying Wei[2,#], Xiaoli Luo[1,#], Yuansheng Liu[1], Kenli Li[1], Qiang Zhang[3,4], Xin Gao[5], Sizhe Yan[2], Xia Wu[2], Xingyue Jiang[2], Mingquan Liu[1], Dongsheng Cao[6], Leyi Wei[7,8], Xiangxiang Zeng[1,*] , Junfeng Shi[2,*]

[1] College of Information Science and Engineering, Hunan University, Changsha, China

[2] State Key Laboratory of Chemo/Bio-Sensing and Chemometrics, School of Biomedical Sciences, Hunan University, Changsha, China

[3] ZJU-Hangzhou Global Scientific and Technological Innovation Center, Hangzhou, 311200, China

[4] College of Computer Science and Technology, Zhejiang University, Hangzhou, 310013, China

[5] Computational Bioscience Research Center (CBRC), Computer, Electrical and Mathematical Sciences and Engineering Division, King Abdullah University of Science and Technology (KAUST), Thuwal, Saudi Arabia

[6] Xiangya School of Pharmaceutical Sciences, Central South University, Changsha, China

[7] School of Software, Shandong University, Jinan, China

[8] Joint SDU-NTU Centre for Artificial Intelligence Research (C-FAIR), Shandong University, Jinan, China

[#] These authors contributed equally.

*corresponding authors: xzeng@hnu.edu.cn, Jeff-Shi@hnu.edu.cn.

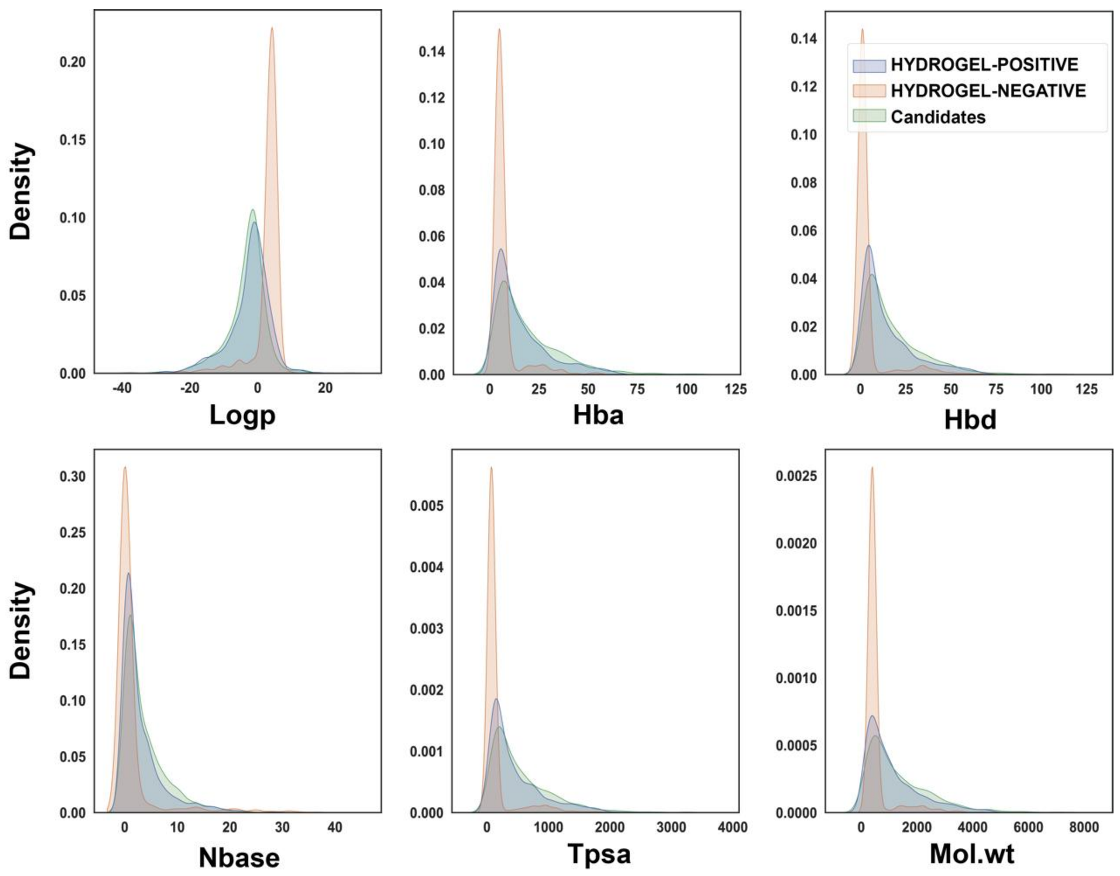

Figure S1. The property distributions of these three clusters. Quantitative analysis of properties is in Supplementary Tables 4-9.

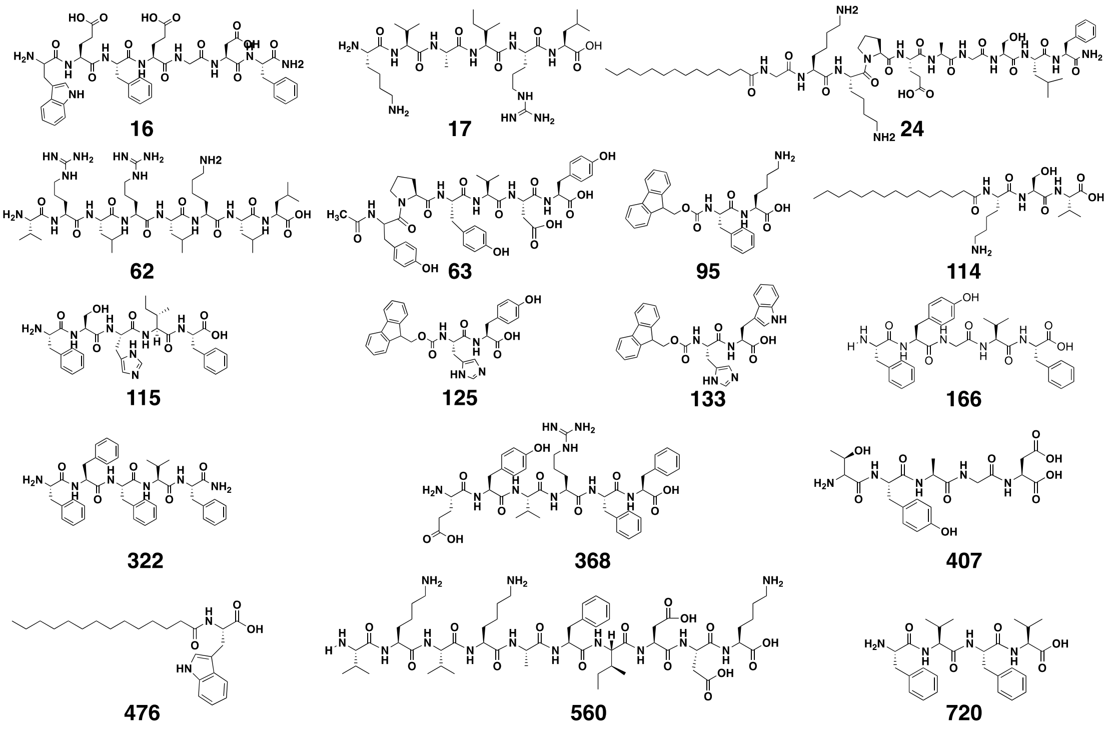

Figure S2. Chemical structures and identification numbers (IDN) of peptides selected from the candidate library.

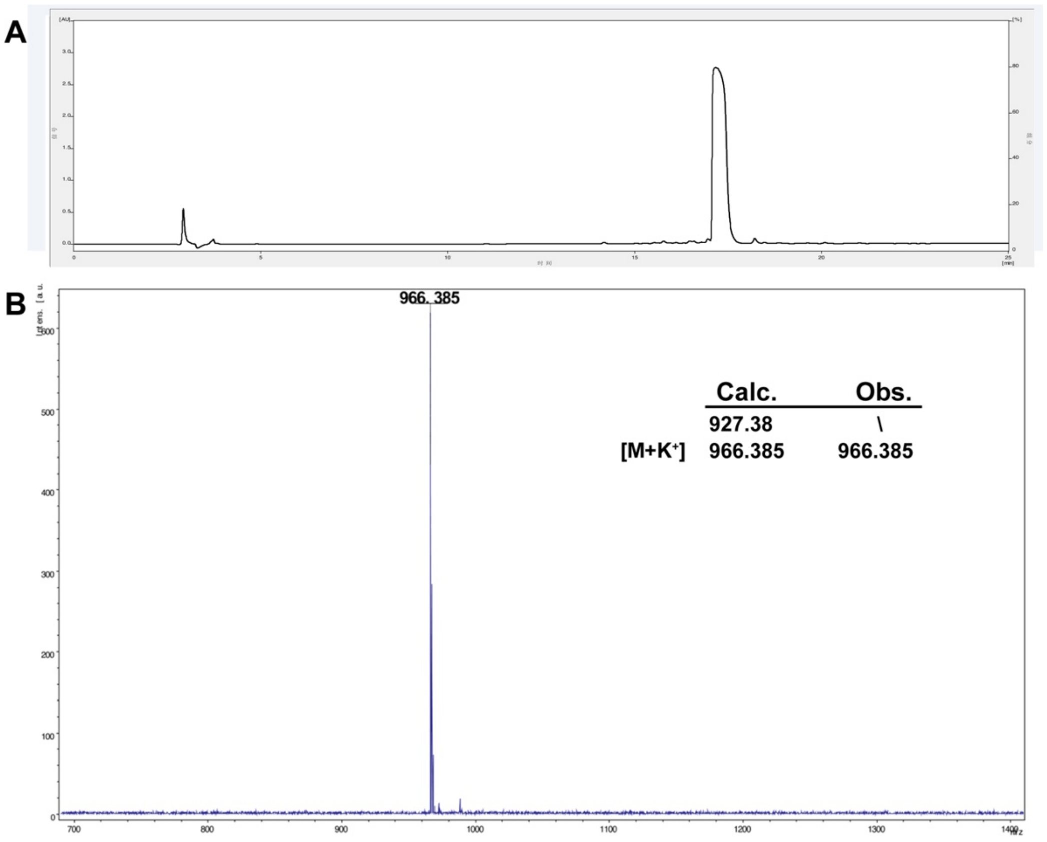

Figure S3. (A) Analytical HPLC and (B) MS spectra of molecule **16**.

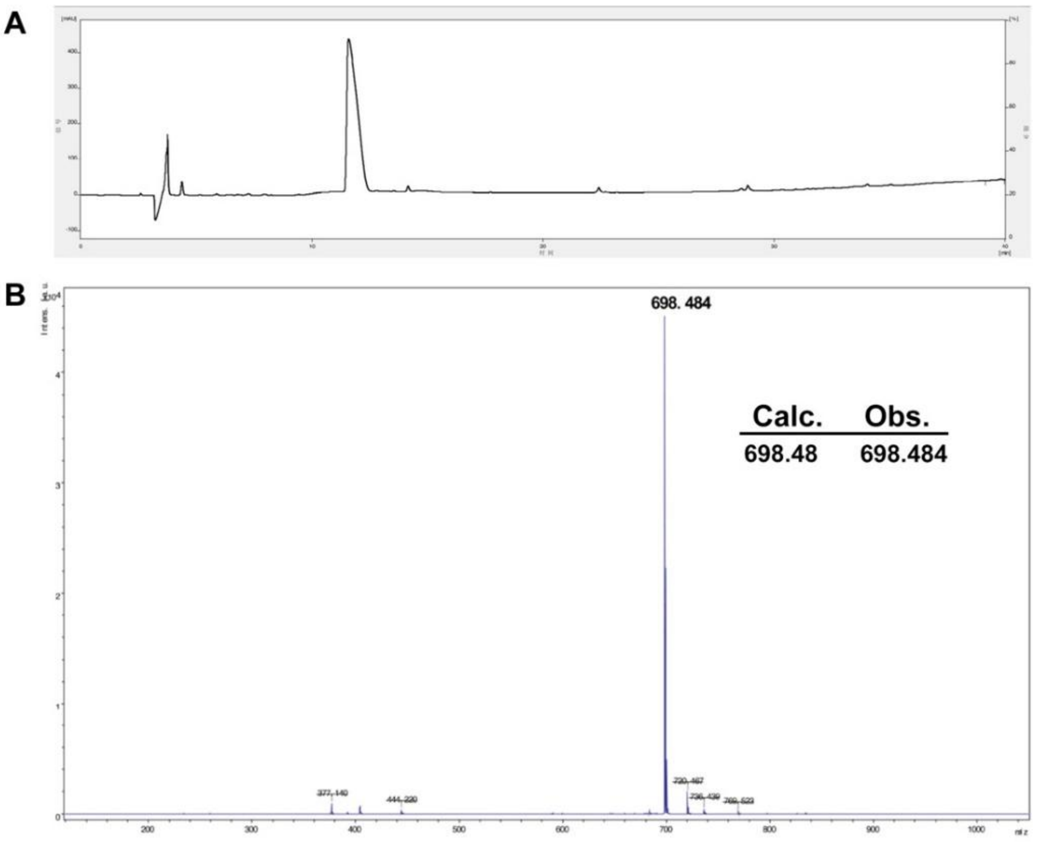

Figure S4. (A) Analytical HPLC and (B) MS spectra of molecule **17**.

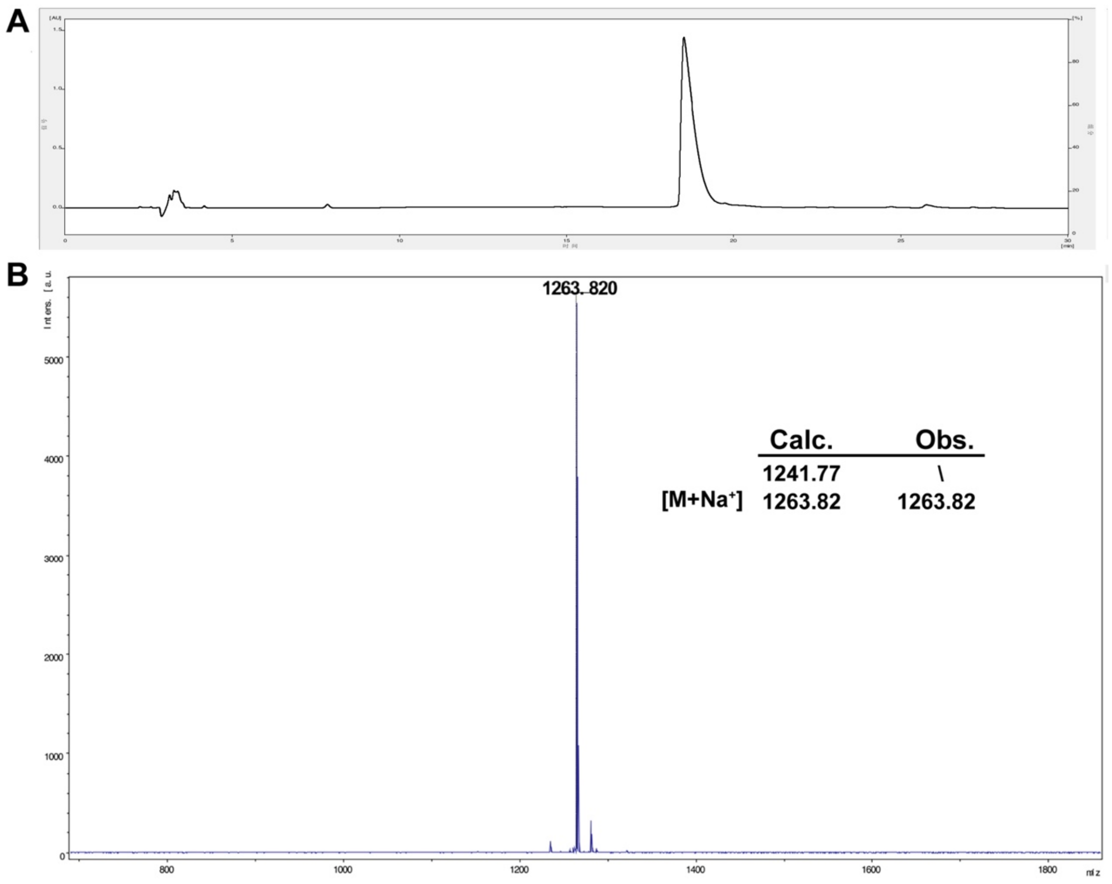

Figure S5. (A) Analytical HPLC and (B) MS spectra of molecule **24**.

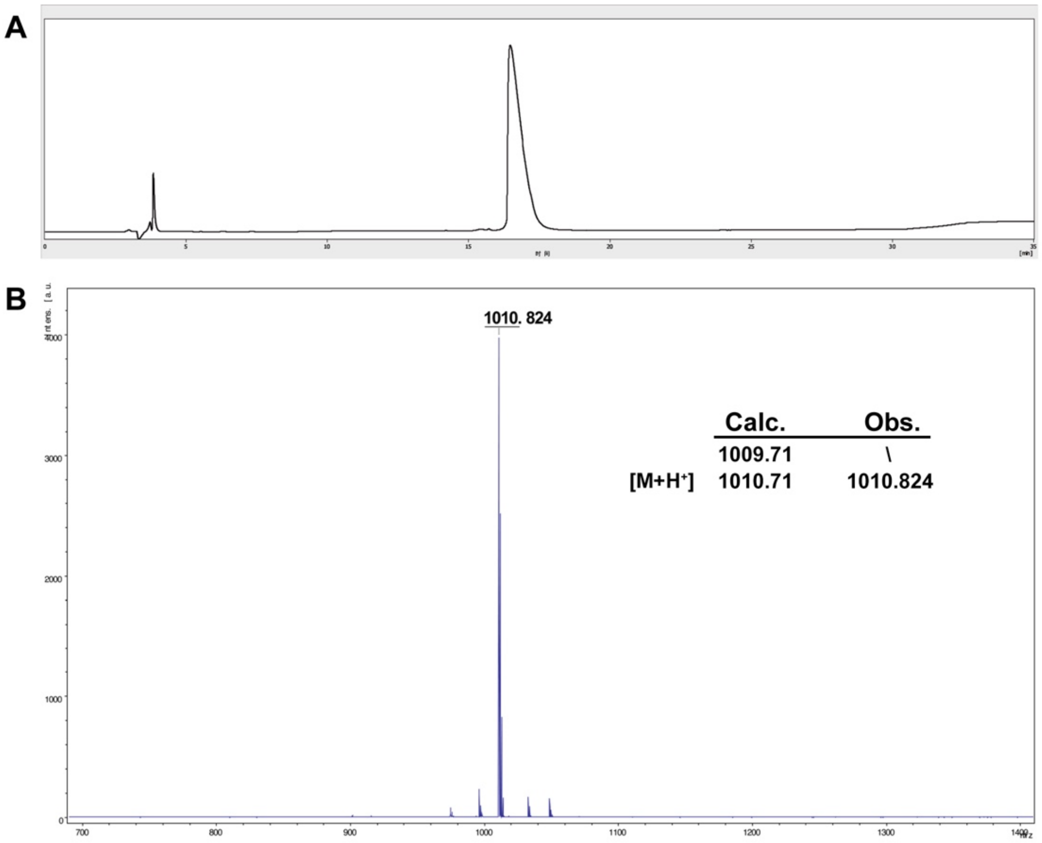

Figure S6. (A) Analytical HPLC and (B) MS spectra of molecule **62**.

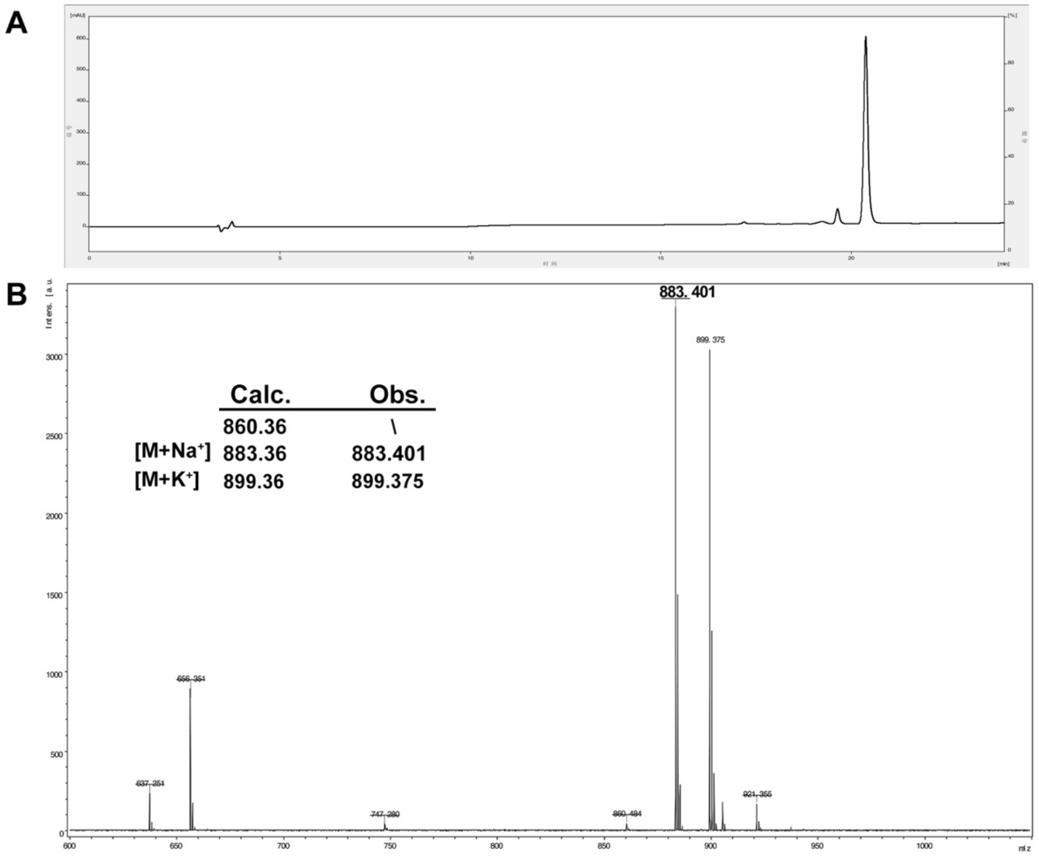

Figure S7. (A) Analytical HPLC and (B) MS spectra of molecule **63**.

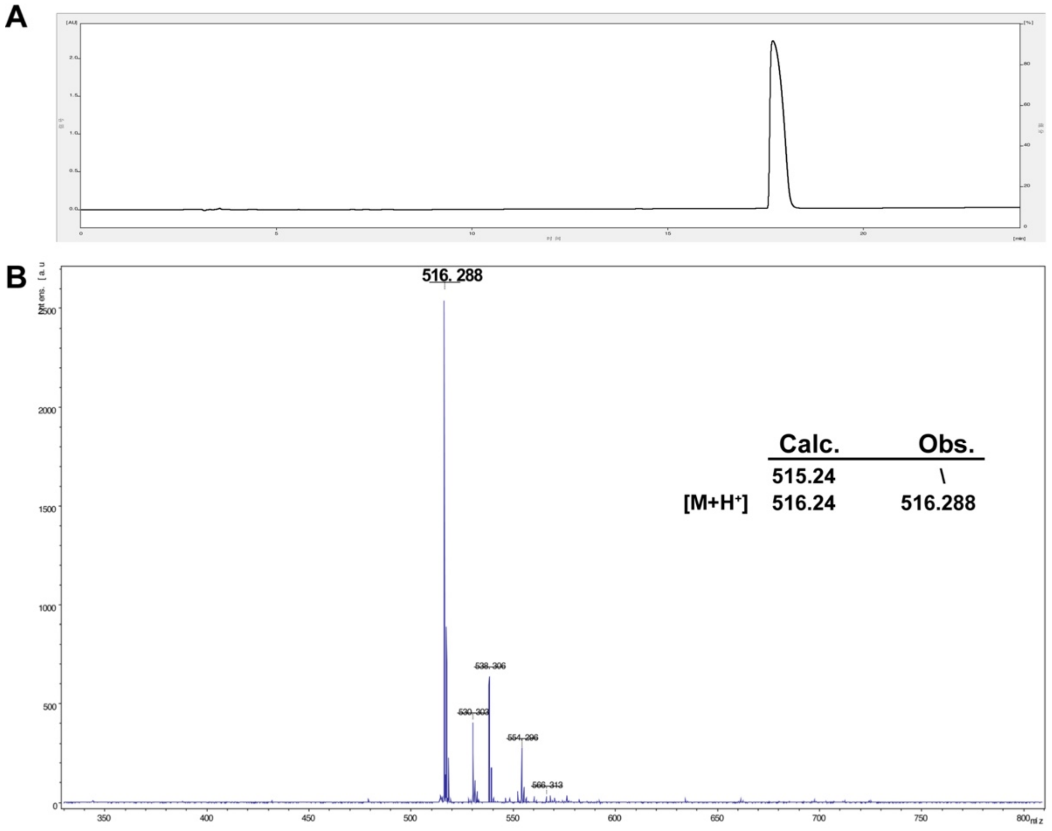

Figure S8. (A) Analytical HPLC and (B) MS spectra of molecule **95**.

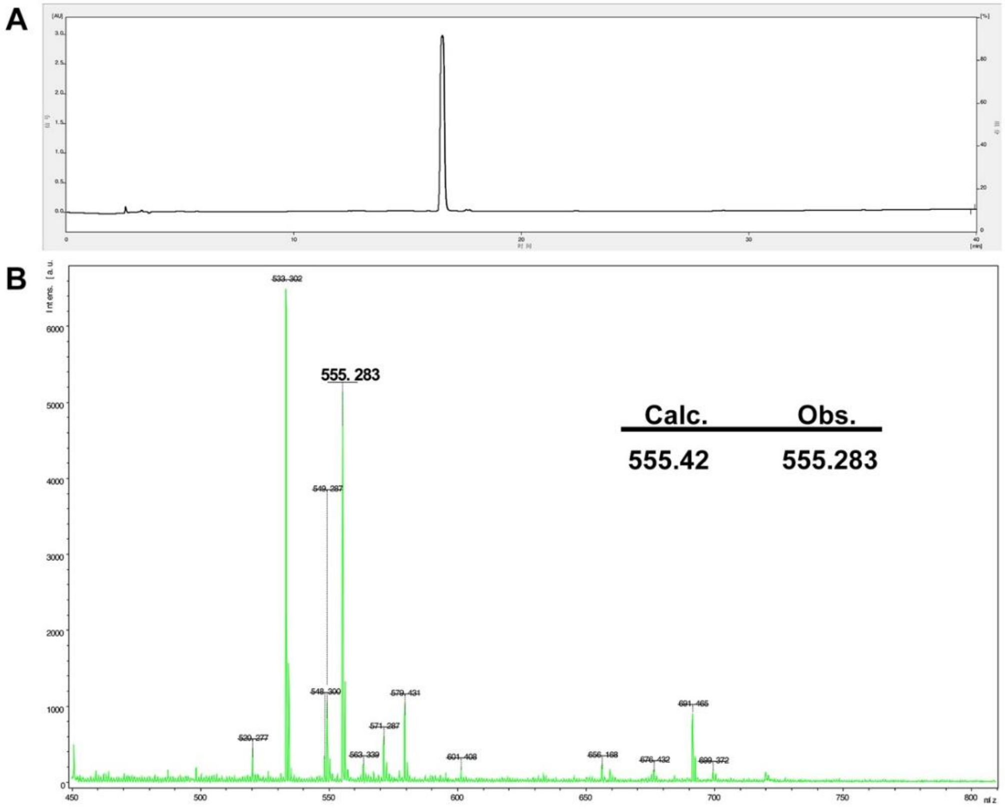

Figure S9. (A) Analytical HPLC and (B) MS spectra of molecule **114**.

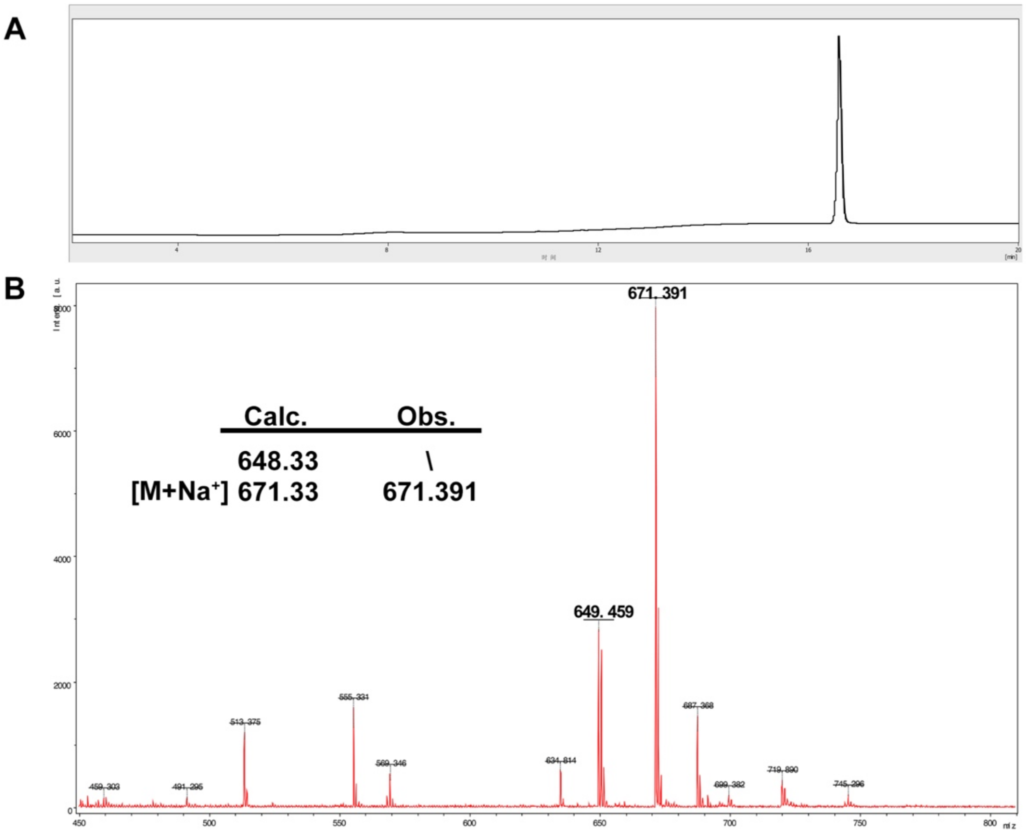

Figure S10. (A) Analytical HPLC and (B) MS spectra of molecule **115**.

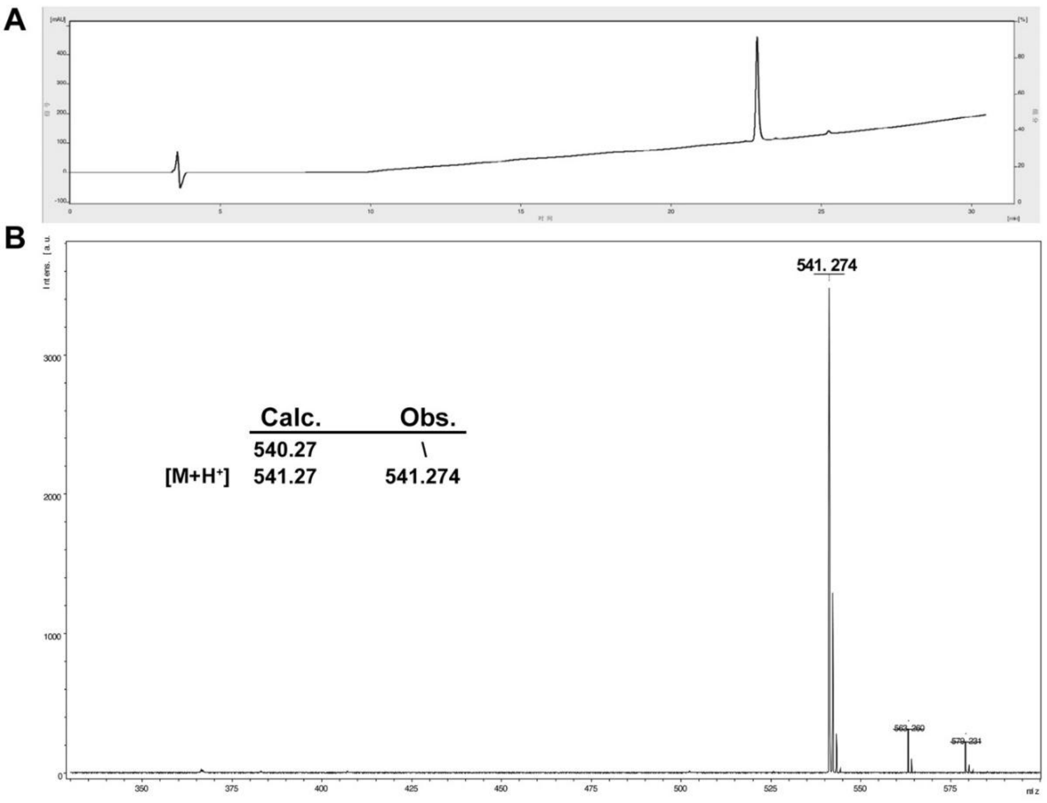

Figure S11. (A) Analytical HPLC and (B) MS spectra of molecule **125**.

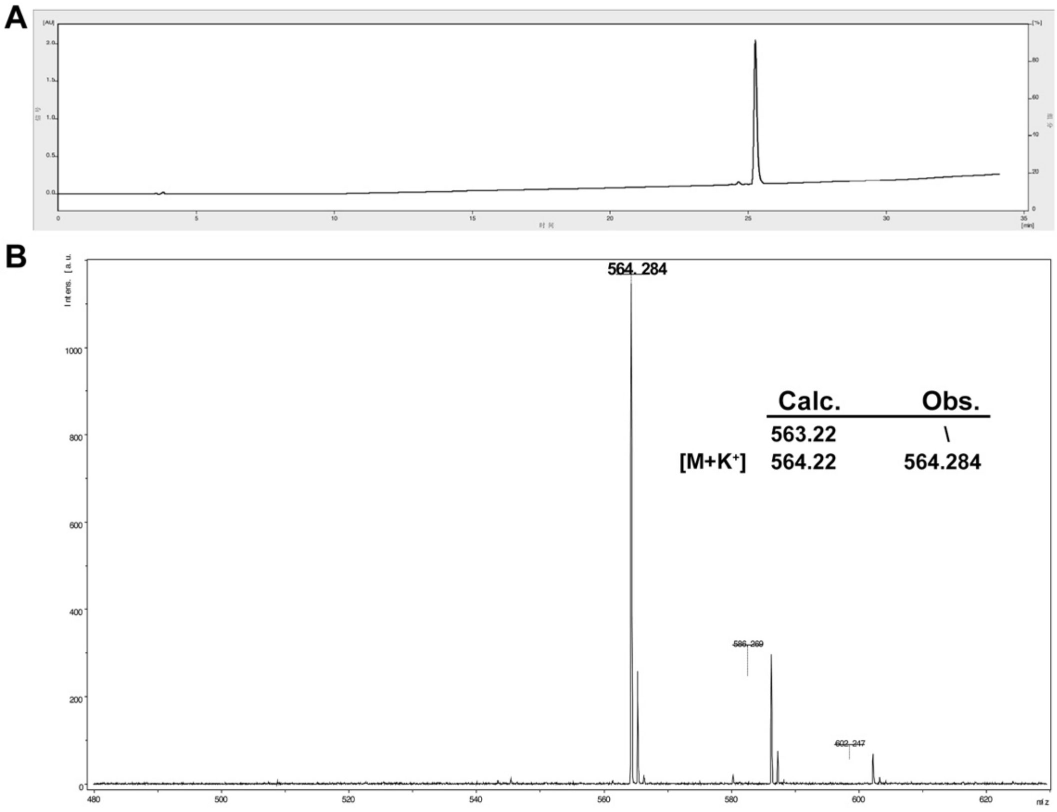

Figure S12. (A) Analytical HPLC and (B) MS spectra of molecule **133**.

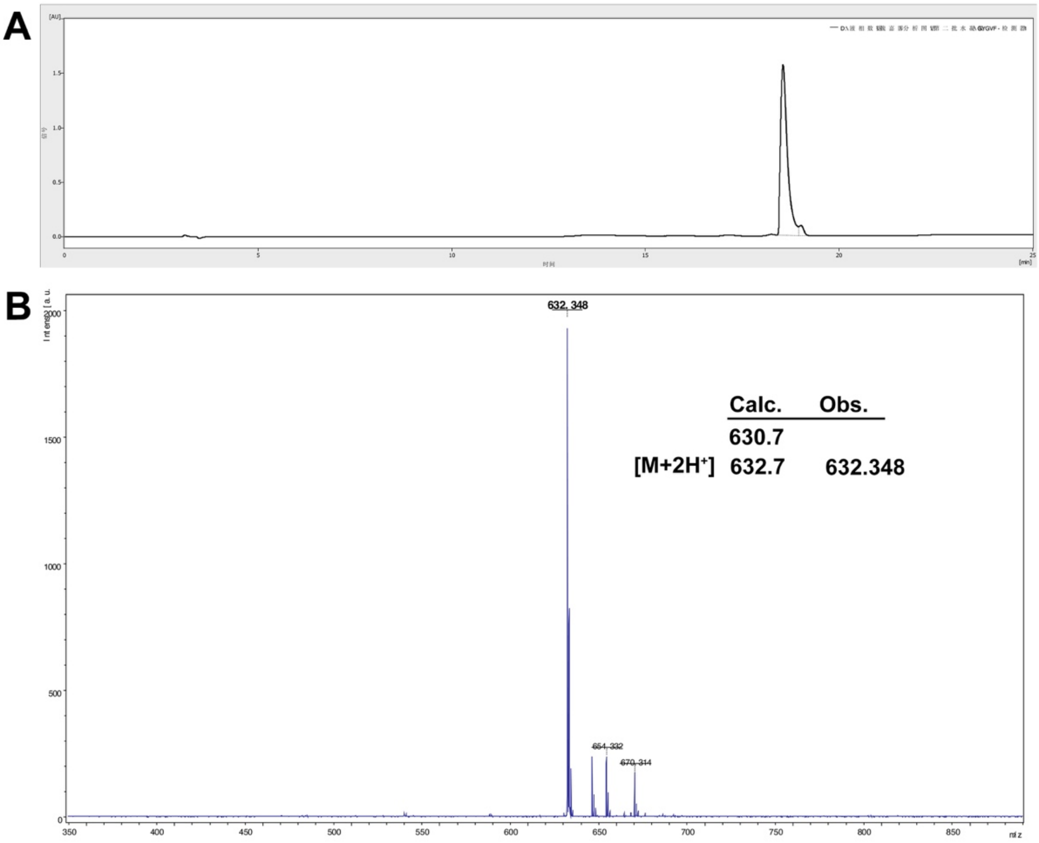

Figure S13. (A) Analytical HPLC and (B) MS spectra of molecule **166**.

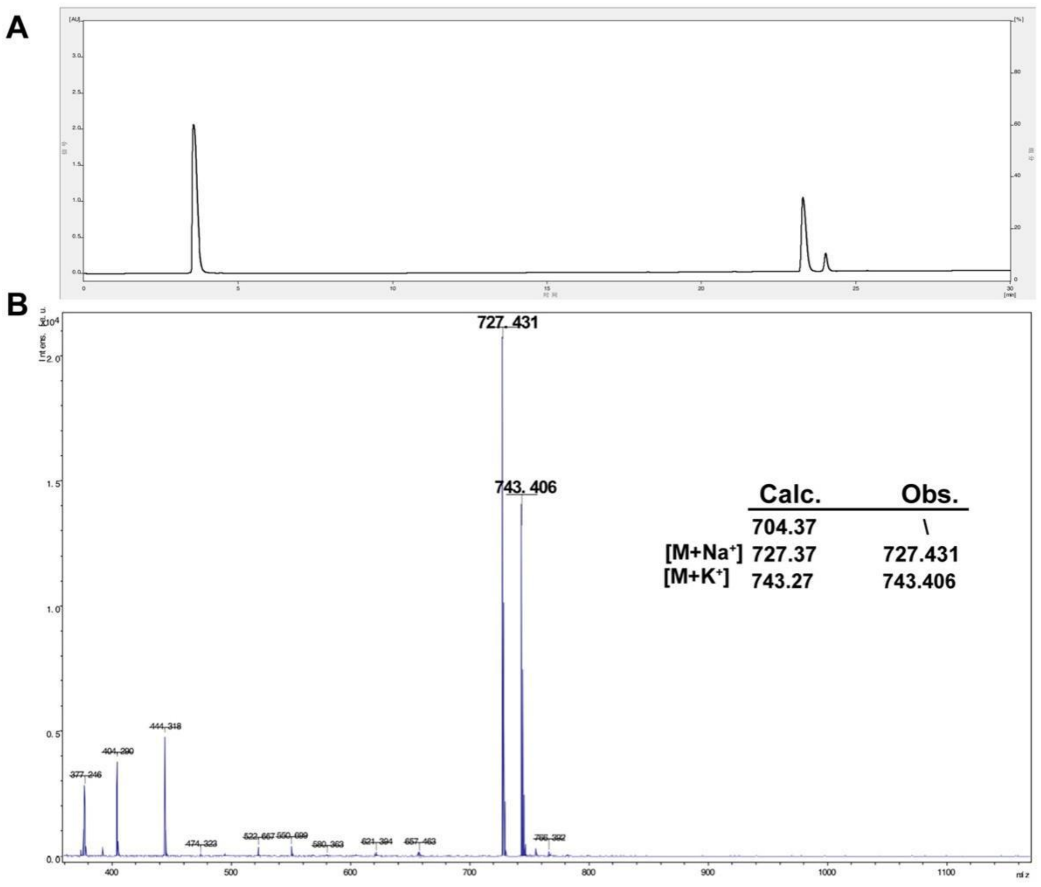

Figure S14. (A) Analytical HPLC and (B) MS spectra of molecule **322**.

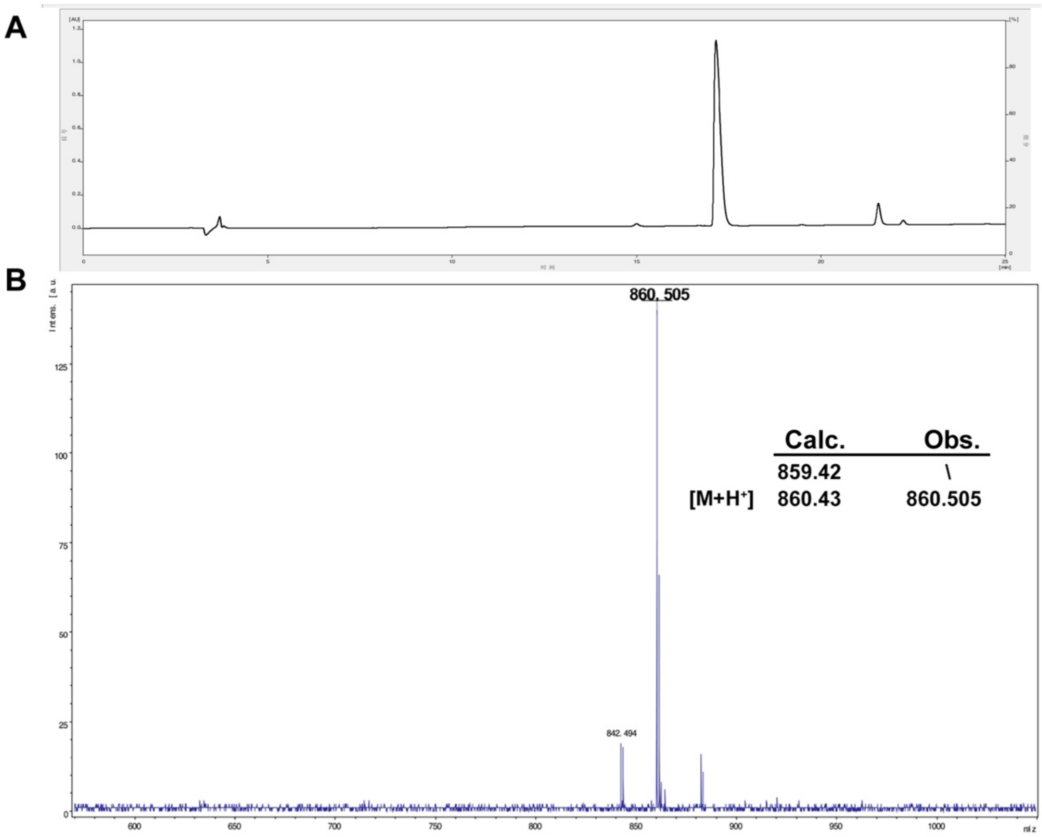

Figure S15. (A) Analytical HPLC and (B) MS spectra of molecule **368**.

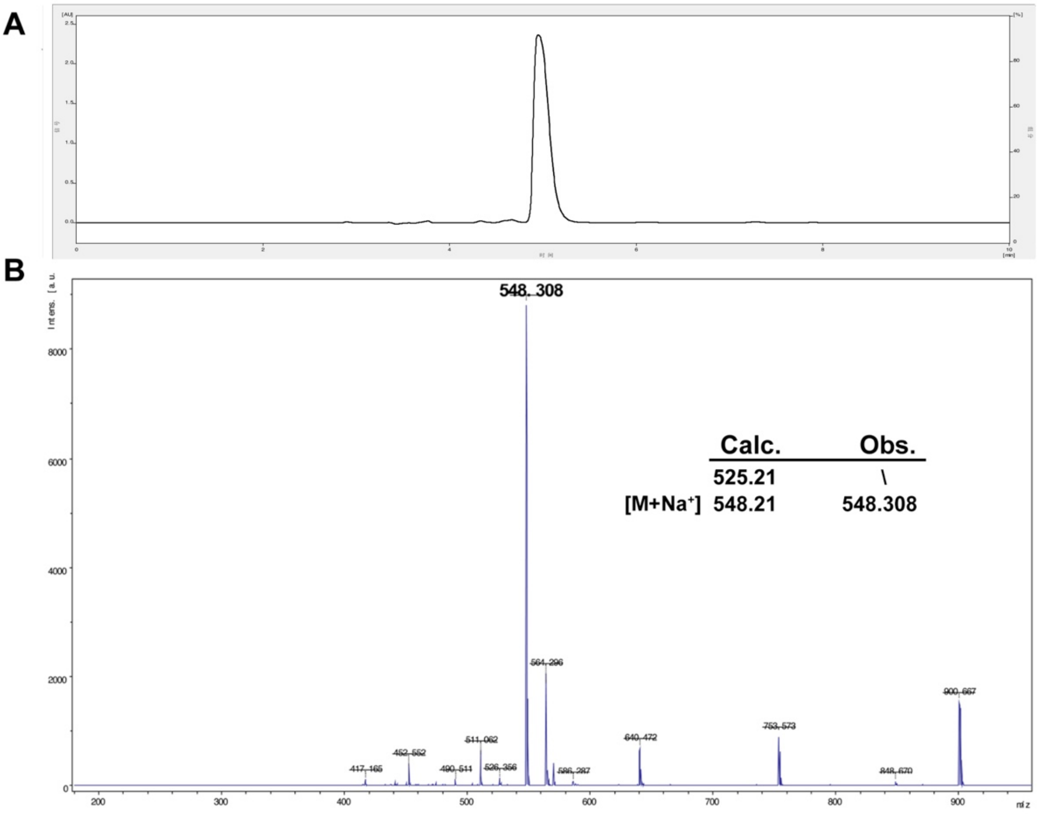

Figure S16. (A) Analytical HPLC and (B) MS spectra of molecule **407**.

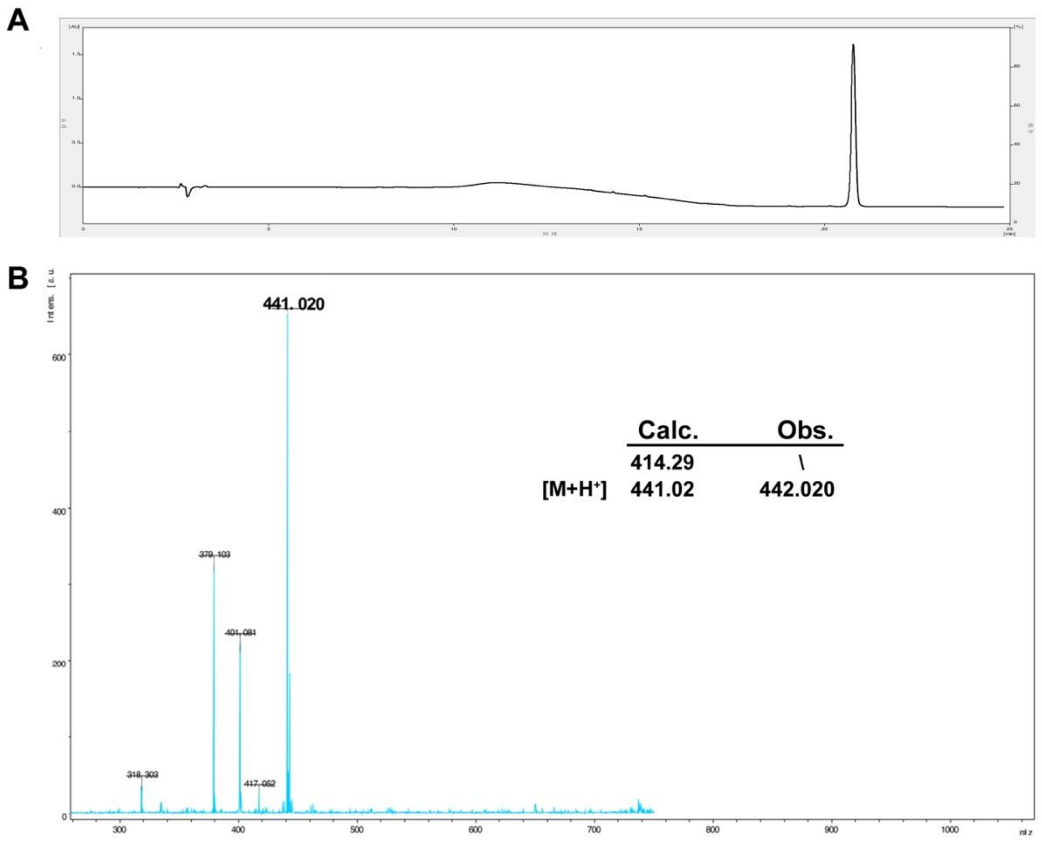

Figure S17. (A) Analytical HPLC and (B) MS spectra of molecule **476**.

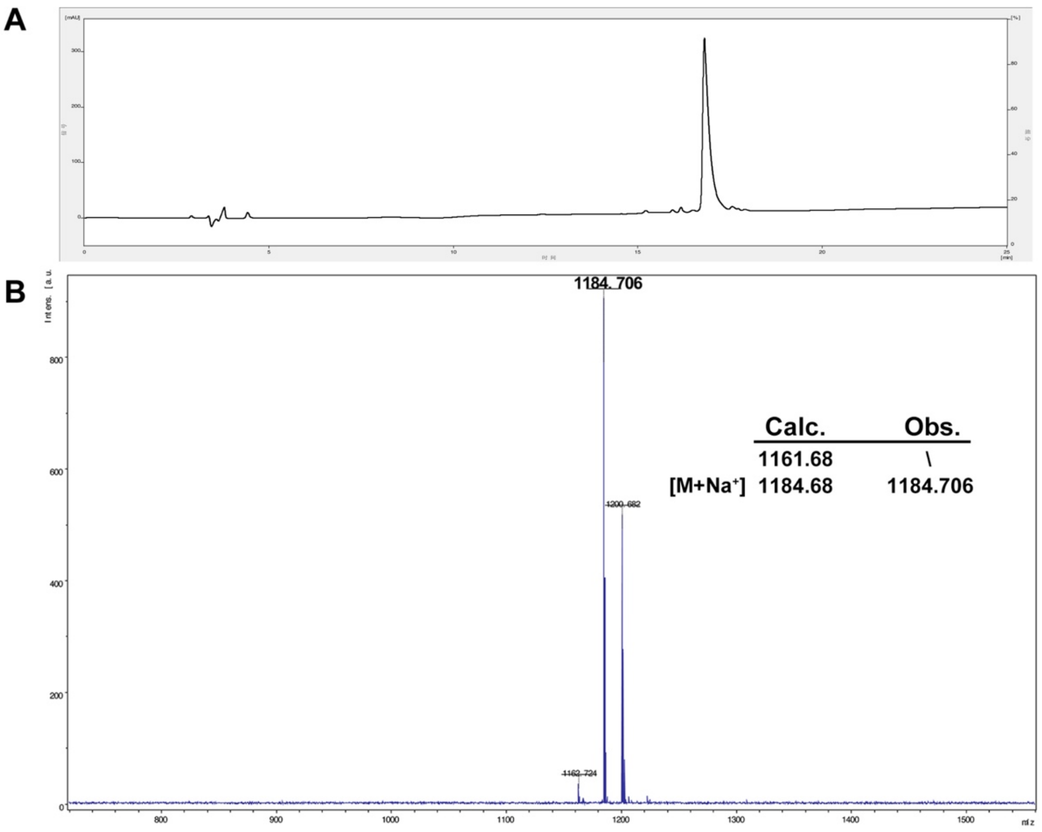

Figure S18. (A) Analytical HPLC and (B) MS spectra of molecule **560**.

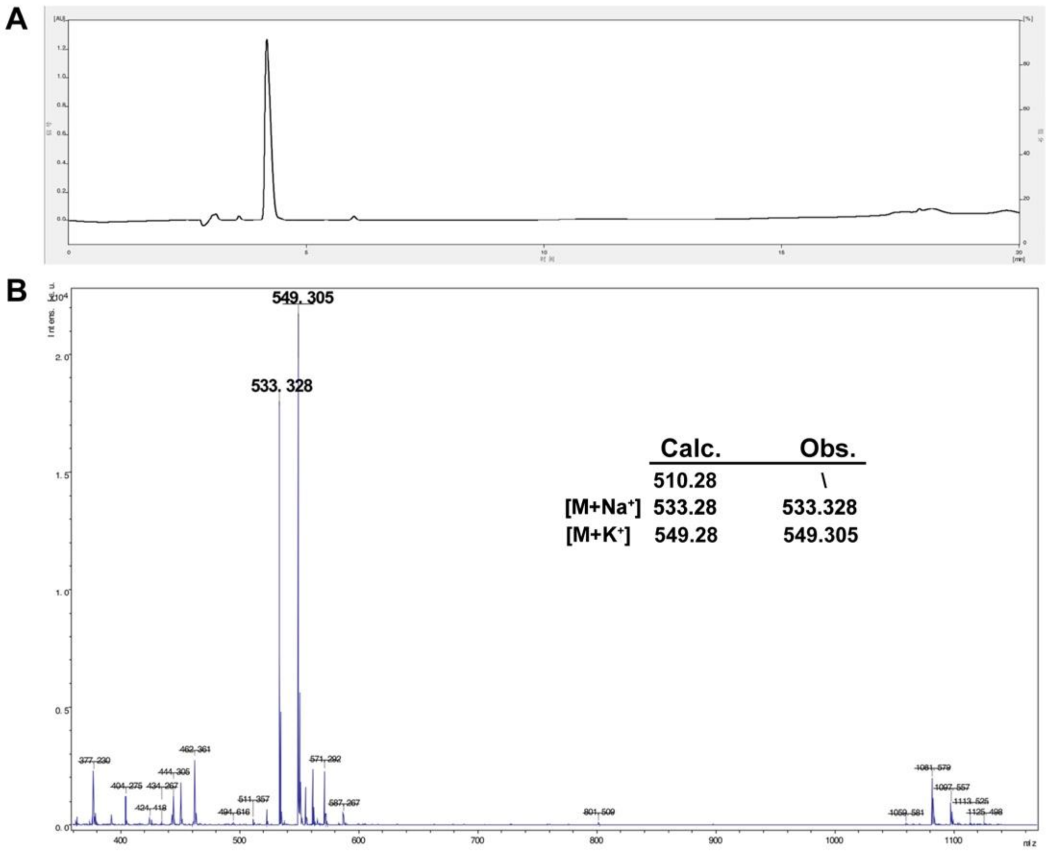

Figure S19. (A) Analytical HPLC and (B) MS spectra of molecule **720**.

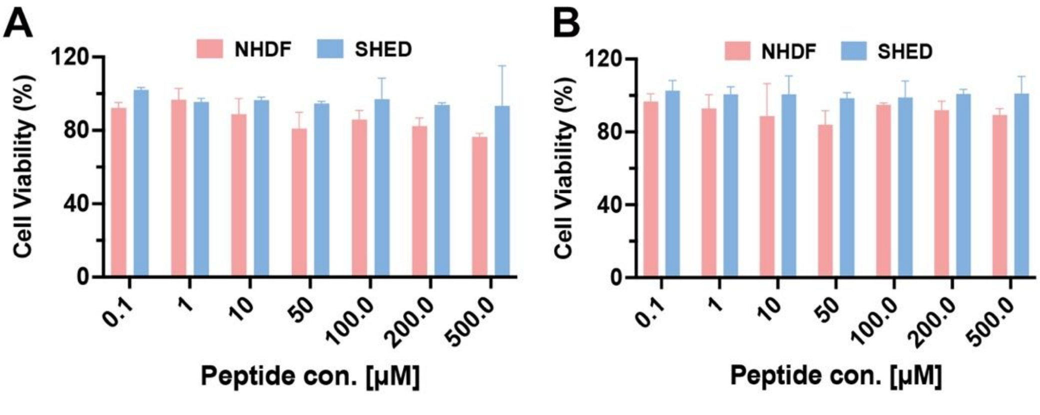

Figure S20. Cytotoxicity of peptides 16 (A) and 62 (B) towards NHDF and SHED cells at concentrations ranging from 0.1-500μM.

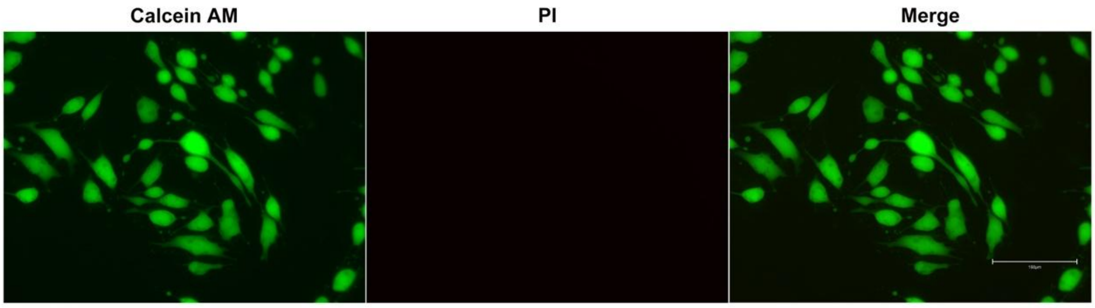

Figure S21. Live/dead assays of SHED cells seeded on standard tissue-culture plates.

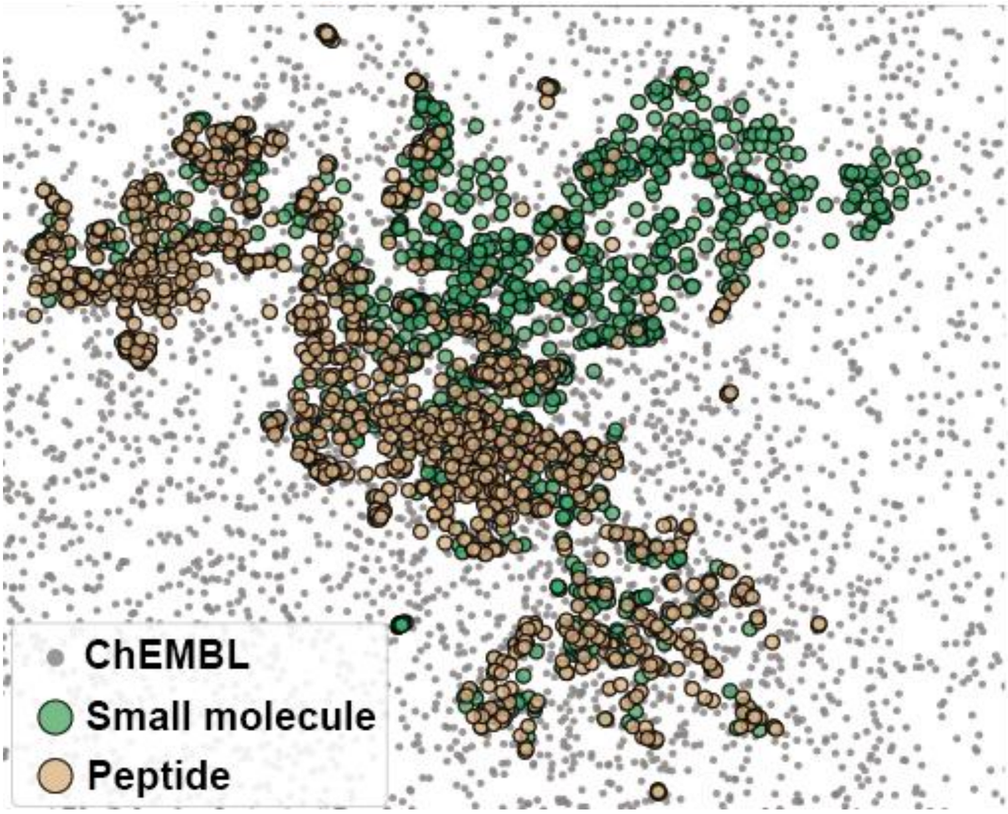

Figure S22. UMAP visualization of chemical space distribution of small molecules and peptides in the training set.

Table S1. Chemical properties of selected molecules.

IDN	Sequences	Molecular Weight	Theoretical pI
**17**	KVAIRL-NH_2	697.49	11.48
**24**	$CH_3(CH2)_{12}$-GKKPEAGSLF-NH_2	1241.77	12.41
**63**	Ac-YPYVDV	843.27	3.12
**114**	$CH_3(CH2)_{12}$-KSV	555.42	10.09
**115**	FSHIF-NH_2	648.33	7.88
**125**	Fmoc-HY	540.20	7.88
**407**	TYAGD	525.20	3.12
**720**	FVFV	524.29	7.00

Table S2. HydrogelFinder-predict Model training data.

Datasets	Training set	Testing set
HYDROGEL-POSITIVE	2,402	267
HYDROGEL-NEGATIVE	15,497	1,722

Table S3. Number of modifiers in datasets.

Datasets	Ac	Fmoc	Nap	Lipidation	S-S
HYDROGEL-POSITIVE	67	22	7	16	47
Peptides-based Candidates	16	5	2	5	0

Table S4. Quantifying the area of overlap of logp properties.

Datasets	HYDROGEL-POSITIVE	HYDROGEL-NEGATIVE	Candidates
HYDROGEL-POSITIVE	1	0.37	0.89
HYDROGEL-NEGATIVE	0.37	1	0.29
Candidates	0.89	0.29	1

Table S5. Quantifying the area of overlap of Hba properties.

Datasets	HYDROGEL-POSITIVE	HYDROGEL-NEGATIVE	Candidates
HYDROGEL-POSITIVE	1	0.55	0.89
HYDROGEL-NEGATIVE	0.55	1	0.45
Candidates	0.89	0.45	1

Table S6. Quantifying the area of overlap of Hbd properties.

Datasets	HYDROGEL-POSITIVE	HYDROGEL-NEGATIVE	Candidates
HYDROGEL-POSITIVE	1	0.44	0.88
HYDROGEL-NEGATIVE	0.44	1	0.36
Candidates	0.88	0.36	1

Table S7. Quantifying the area of overlap of Nbase properties.

Datasets	HYDROGEL-POSITIVE	HYDROGEL-NEGATIVE	Candidates
HYDROGEL-POSITIVE	1	0.65	0.89
HYDROGEL-NEGATIVE	0.65	1	0.56
Candidates	0.89	0.56	1

Table S8. Quantifying the area of overlap of Tpsa properties.

Datasets	HYDROGEL-POSITIVE	HYDROGEL-NEGATIVE	Candidates
HYDROGEL-POSITIVE	1	0.45	0.89

HYDROGEL-NEGATIVE	0.45	1	0.36
Candidates	0.89	0.36	1

Table S9. Quantifying the area of overlap of Mol.wt properties.

Datasets	HYDROGEL-POSITIVE	HYDROGEL-NEGATIVE	Candidates
HYDROGEL-POSITIVE	1	0.49	0.91
HYDROGEL-NEGATIVE	0.49	1	0.41
Candidates	0.91	0.41	1

Random Sampling Method

The random sampling mentioned in this article is implemented using the “shuffle” function provided by the “random” module of the python standard library. Specifically, random. shuffle() uses a random number generator to shuffle the elements in the sequence, so each call to it produces a different result. However, we have set random seed here to ensure the repeatability of the random extraction of the experiment.

RDKit Data Filtering

Filtering molecular data and checking whether a molecule can be converted to a graph using RDKit is divided into the following 3 steps：

Step 1: Importing RDKit Library. We began by importing the RDKit library, a powerful tool for cheminformatics and molecular informatics.

Step 2: Loading Molecular Data. We loaded molecular data into RDKit. The molecular data can be represented using SMILES (Simplified Molecular Input Line Entry System) notation or other supported molecular file formats.

Step 3: Checking Molecular Validity. After loading the molecule using Chem.MolFromSmiles() or similar RDKit functions, we checked whether the molecule was valid and could be converted into a graph.

High-throughput Prediction HydrogelFinder-predict Model

Support vector machine (SVM) belongs to supervised learning methods. It is a widely used machine learning algorithm for binary classification tasks. In our experiments, we are using the radial basis function (RBF). For the SVM models, the parameter optimization was performed using grid search. The model with C = 10 and γ = 0.01 was considered to have the highest AUROC (0.9862) towards the testing set of the HYDROGEL dataset.

We carefully selected relevant molecular features and descriptors for input to the SVM model. The model was trained to discriminate active compounds that could self-assemble to form hydrogels from inactive ones according to their 2,048-bit-radius extended connectivity fingerprint (ECFP) representations. We split the dataset into training and test sets in the number 9:1 to train the model (Supplementary Table 2).

The evaluation metrics used Receiver Operating Characteristic (ROC) Curve. We plotted the ROC curve and calculated the area under the ROC curve (AUROC) to assess the model's discriminatory power, where the AUROC is calculated as follows:

$$AUROC = \frac{\sum(p_i, n_i)_{p_i > n_i}}{P * N},$$

where P is the number of positive samples, N is the number of negative samples, p_i is the positive sample prediction score, and n_i is the negative sample prediction score.

We set the threshold to 0.5, which is the default threshold for binary classification tasks in machine learning. At this threshold, the accuracy of the model is 99.56%. The formula for calculating the accuracy rate is as follows:

$$Accuracy = \frac{TP+TN}{TP+FN+FP+TN},$$

where TP is predicted to be a positive sample and is actually a positive sample, TN is predicted to be a negative sample and is actually a negative sample, FN is predicted to be a negative sample and is actually a positive sample, FP is predicted to be a positive sample and actually a negative sample.

Calculate the area of overlap of kernel density maps

We used Simpson's law (simps function) to calculate the overlap area of the kernel density maps. Specifically, for two kernel density estimation curves, $f(x)$ and $g(x)$, we aim to determine their overlap area using the formula:

$$overlap\ area\ = \int [a, b]\ min(f(x), g(x))\ dx\ ,$$

where $[a, b]$ represents the region of intersection of the two curves, and $min(f(x), g(x))$ signifies selecting the smaller value of the two curves at each x point.

Simpson's law estimates this overlap area by discretizing this integral. First, the interval $[a, b]$ is divided into small intervals, $min(f(x), g(x))$ is then computed within each of these intervals, and finally, the area over these small intervals is accumulated.

Up-sampling strategy

In addressing the imbalance between positive and negative samples in a dataset, an upsampling strategy is employed. This method is crucial for improving the performance of machine learning models by balancing the class distribution. The Python pandas library is utilized for data manipulation, and sklearn.utils libraries is leveraged for performing the upsampling. Specifically, the resample function in the sklearn.utils libraries allow us to adjust the number of samples in a class by repeating instances. Thus, the final ratio of positive and negative samples used in train HydrogelFinder-predict was 15728:15497.
